# Consumption of penicillins in the community, European Union/European Economic Area, 1997–2017

**DOI:** 10.1093/jac/dkab173

**Published:** 2021-08-01

**Authors:** Robin Bruyndonckx, Niels Adriaenssens, Niel Hens, Ann Versporten, Dominique L Monnet, Geert Molenberghs, Herman Goossens, Klaus Weist, Samuel Coenen, the ESAC-Net study group, Reinhild Strauss, Eline Vandael, Stefana Sabtcheva, Marina Payerl-Pal, Isavella Kyriakidou, Jiří Vlček, Ute Wolff Sönksen, Elviira Linask, Emmi Sarvikivi, Philippe Cavalié, Marc Schneider, Flora Kontopidou, Ria Benkő, Gudrun Aspelund, Ajay Oza, Filomena Fortinguerra, Ieva Rutkovska, Jolanta Kuklytė, Marcel Bruch, Peter Zarb, Stephanie Natsch, Hege Salvesen Blix, Anna Olczak-Pieńkowska, Ana Silva, Gabriel Adrian Popescu, Tomáš Tesař, Milan Čižman, Mayte Alonso Herreras, Vendela Bergfeldt, Berit Müller-Pebody

**Affiliations:** 1 Laboratory of Medical Microbiology, Vaccine & Infectious Disease Institute (VAXINFECTIO), University of Antwerp, Antwerp, Belgium; 2 Interuniversity Institute for Biostatistics and statistical Bioinformatics (I-BIOSTAT), Data Science Institute, Hasselt University, Hasselt, Belgium; 3 Centre for General Practice, Department of Family Medicine & Population Health (FAMPOP), University of Antwerp, Antwerp, Belgium; 4 Centre for Health Economic Research and Modelling Infectious Diseases, Vaccine & Infectious Disease Institute (VAXINFECTIO), University of Antwerp, Belgium; 5 Disease Programmes Unit, European Centre for Disease Prevention and Control, Stockholm, Sweden; 6 Interuniversity Institute for Biostatistics and statistical Bioinformatics (I-BIOSTAT), Catholic University of Leuven, Leuven, Belgium

## Abstract

**Objectives:**

Data on consumption of penicillins in the community were collected from 30 EU/European Economic Area (EEA) countries over two decades. This article reviews temporal trends, seasonal variation, presence of change-points and changes in the composition of the main subgroups of penicillins.

**Methods:**

For the period 1997–2017, data on consumption of penicillins, i.e. β-lactam antibacterials, penicillins (ATC group J01C), in the community aggregated at the level of the active substance, were collected using the WHO ATC/DDD methodology (ATC/DDD index 2019). Consumption was expressed in DDD per 1000 inhabitants per day and in packages per 1000 inhabitants per day. Consumption of penicillins was analysed based on ATC-4 subgroups, and presented as trends, seasonal variation, presence of change-points and compositional changes.

**Results:**

In 2017, consumption of penicillins in the community expressed in DDD per 1000 inhabitants per day varied by a factor of 4.9 between countries with the highest (Spain) and the lowest (the Netherlands) consumption. An increase in consumption of penicillins, which was not statistically significant, was observed between 1997 and 2003 and up to 2010. A decrease, which was not statistically significant, was observed from 2010 onwards. Proportional consumption of combinations of penicillins, including β-lactamase inhibitors (J01CR) increased during 1997–2017, which coincided with a decrease in the proportional consumption of extended-spectrum penicillins (J01CA) and narrow-spectrum penicillins (J01CE).

**Conclusions:**

Considerable variation in the patterns of consumption of penicillins was observed between EU/EEA countries. The consumption of penicillins in the EU/EEA community did not change significantly over time, while the proportional consumption of combinations of penicillins increased.

## Introduction

This article presents data from the European Surveillance of Antimicrobial Consumption Network (ESAC-Net,^1^ formerly ESAC) on consumption of penicillins for 30 EU/European Economic Area (EEA) countries in 2017 (Table 1). It updates previous ESAC studies published in 2006 and 2011, and in doing so it provides updated comparable and reliable information on antibiotic consumption that can aid in fighting the global problem of antimicrobial resistance.^2^^,^^3^ In 2017, penicillins represented 42.3% of antibiotic consumption in the community.^4^ The objective of this study was to analyse temporal trends, seasonal variation and the presence of change-points in consumption of penicillins in the community (i.e. primary care sector) for the period 1997–2017, as well as to analyse the composition of consumption of penicillins over time.

**Table 1.** Classification of β-lactam antibacterials, penicillins (J01C; ATC/DDD index 2019)

Extended-spectrum penicillins	
J01CA01	Ampicillin
J01CA02	Pivampicillin
J01CA03	*Carbenicillin* ^a^
J01CA04	**Amoxicillin** ^b^
J01CA05	*Carindacillin* ^a^
J01CA06	Bacampicillin
J01CA07	*Epicillin* ^a^
J01CA08	Pivmecillinam
J01CA09	*Azlocillin*
J01CA10	*Mezlocillin*
J01CA11	Mecillinam
J01CA12	Piperacillin
J01CA13	*Ticarcillin*
J01CA14	*Metampicillin* ^a^
J01CA15	*Talampicillin* ^a^
J01CA16	*Sulbenicillin* ^a^
J01CA17	Temocillin^a^
J01CA18	*Hetacillin* ^a^
J01CA19	*Aspoxicillin* ^a^
J01CA20	*Combinations* ^a^
J01CA51	Ampicillin, combinations
Narrow-spectrum penicillins	
J01CE01	Benzylpenicillin
J01CE02	**Phenoxymethylpenicillin** ^b^
J01CE03	*Propicillin*
J01CE04	*Azidocillin* ^a^
J01CE05	Pheneticillin
J01CE06	*Penamecillin*
J01CE07	*Clometocillin*
J01CE08	Benzathine benzylpenicillin
J01CE09	Procaine penicillin
J01CE10	phenoxymethylpenicillin
J01CE30	Combinations
Combinations of penicillins, including β-lactamase inhibitors	
J01CR01	Ampicillin and BLI
J01CR02	**Amoxicillin and BLI** ^b^
J01CR03	*Ticarcillin and BLI*
J01CR04	Sultamicillin
J01CR05	Piperacillin and BLI
J01CR50	Combinations of penicillins
β-Lactamase inhibitors	
J01CG01	*Sulbactam*
J01CG02	*Tazobactam* ^a^
Penicillinase-resistant penicillins	
J01CF01	Dicloxacillin
J01CF02	Cloxacillin
J01CF03	*Meticillin* ^a^
J01CF04	Oxacillin
J01CF05	**Flucloxacillin** ^b^
J01CF06	*Nafcillin* ^c^

BLI, β-lactamase inhibitor; **Bold type** indicates that consumption was part of the top 90% of the community consumption of penicillins (J01C) in 28 EU/EEA countries in 2017; *Italic type* indicates that no consumption of this penicillin was reported in 28 EU/EEA countries in 2017.

a No consumption of this penicillin was reported in 30 EU/EEA countries in 2009.

b Consumption was part of the top 90% of community consumption of penicillins (J01C) in 30 EU/EEA countries in 2009.

c This penicillin was not included in the ATC/DDD index in 2009 and was therefore not reported in 2009.

## Methods

The methods for collecting and analysing the data are described in the introductory article of this series.^4^ In summary, data on consumption of penicillins, i.e. β-lactam antibacterials, penicillins (ATC group J01C, referred to as penicillins in this manuscript) aggregated at the level of the active substance, were collected using the WHO ATC/DDD methodology (ATC/DDD index 2019)^5^ and expressed in DDD per 1000 inhabitants per day. In addition, where data were available, consumption of penicillins was expressed in packages per 1000 inhabitants per day. Penicillins (J01C) are classified in five subgroups. Because β-lactamase inhibitors (J01CG) are given in conjunction with extended-spectrum penicillins (J01CA), we focused on only the four main subgroups: penicillins with extended-spectrum [J01CA; extended-spectrum penicillins (ESP)], β-lactamase-sensitive penicillins [J01CE; narrow-spectrum penicillins (NSP)]; β-lactamase-resistant penicillins [J01CF; penicillinase-resistant penicillins (PRP)], and combinations of penicillins including β-lactamase inhibitors [J01CR; combinations of penicillins (COP)].

There are 46 unique ATC codes for penicillins in the ATC/DDD index 2019. Compared with previous descriptions of the consumption of penicillins in the community, two additional substances, i.e. nafcillin (J01CF06) and aspoxicillin (J01CA19), have been assigned an ATC code by the WHO (Table 1).^2^

The evolution of the number of DDD per package over time was assessed using a linear mixed model. The temporal trend, seasonal variation and presence of change-points in consumption of penicillins were assessed using a non-linear change-point mixed model fitted to quarterly data expressed in DDD per 1000 inhabitants per day from 1997 to 2017.^6^ The relative proportions of the main subgroups were assessed through a compositional data analysis modelling yearly data expressed in DDD per 1000 inhabitants per day from 1997 to 2017.^7^

## Results

An overview of consumption of penicillins (ATC J01C) in the community, expressed in DDD and packages per 1000 inhabitants per day for all participating countries between 1997 and 2017 is available as Supplementary data at *JAC* Online (Tables S1 and S2, respectively).

### Consumption of penicillins in the community in 2017

In 2017, four substances accounted for 90% of the consumption of penicillins in the community expressed in DDD per 1000 inhabitants per day: amoxicillin/clavulanic acid (45.9% in 2017 compared with 42.4% in 2009), amoxicillin (34.8% in 2017 compared with 34.7% in 2009), phenoxymethylpenicillin (9% in 2017 compared with 12.9% in 2009) and flucloxacillin (3.2% in 2017 compared with 2.5% in 2009) (Table 1).

**Figure 1.**
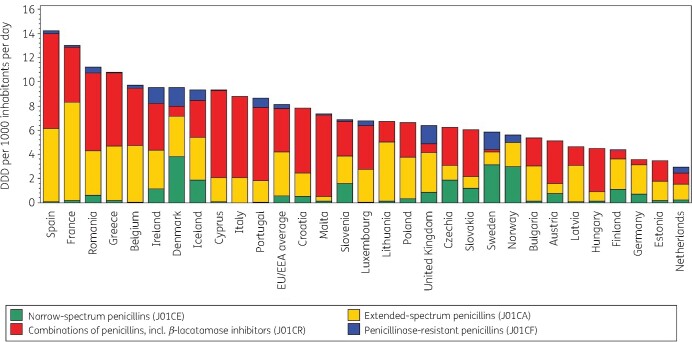
Consumption of penicillins (ATC J01C) in the community, expressed in DDD (ATC/DDD index 2019) per 1000 inhabitants per day, 30 EU/EEA countries, 2017. For Czechia, 2015 data are used. For Slovakia, 2016 data are used. For Cyprus and Romania, total care data, i.e. community and hospital sector combined, are used.

Figure 1 shows the consumption of penicillins in the community subdivided in the four main subgroups expressed in DDD per 1000 inhabitants per day for 30 EU/EEA countries in 2017. Consumption of penicillins in the community varied by a factor of 4.9 between the countries with the highest (14.23 DDD per 1000 inhabitants per day in Spain) and the lowest (2.92 DDD per 1000 inhabitants per day in the Netherlands) consumption in 2017, which was higher than in 2009 (factor of 3.6, from 10.91 DDD per 1000 inhabitants per day in France to 3.03 DDD per 1000 inhabitants per day in Estonia).

In 2017, NSP represented 10.1% (compared with 15.1% in 2009) of penicillin consumption in the community. Large variations in NSP consumption were found, ranging from 3.8 DDD per 1000 inhabitants per day in Denmark to 0.0006 DDD per 1000 inhabitants per day in Italy. Phenoxymethylpenicillin, commonly known as penicillin V, was the most widely reported NSP in most countries. It represented >50% of penicillin consumption in the community in Sweden (53.8%) and Norway (53.5%), but represented <1% of penicillin consumption in Belgium, Cyprus (total care data, i.e. community and hospital sector combined), Greece, Luxembourg, the Netherlands and Spain, with no consumption reported in Italy, Portugal and Slovenia. A wide variety of other NSPs was reported, which varied depending on the countries. For example, benzathine phenoxymethylpenicillin was mainly consumed in Austria, Croatia, Germany and Slovenia, and benzylpenicillin in Greece and Romania (total care data). Pheneticillin was exclusively consumed in the Netherlands, and procaine benzylpenicillin was exclusively consumed in Spain.

In 2017, ESP represented 38.6% (compared with 37.8% in 2009) of penicillin consumption in the community. ESP consumption ranged from 8.2 DDD per 1000 inhabitants per day in France to 0.37 DDD per 1000 inhabitants per day in Malta. Amoxicillin was by far the most commonly reported ESP. It represented >50% of penicillin consumption in the community in Lithuania (72.2%), Germany (68.4%), Latvia (64.0%), France (62.0%), Bulgaria (53.7%), Poland (52.4%) and the United Kingdom (51.2%). Malta had the lowest proportional consumption (5.0%). Pivmecillinam was commonly reported in Denmark (26.2%), Norway (23.2%), Finland (14.0%) and Iceland (13.0%). Mecillinam was only reported in Greece and Norway; pivampicillin was only reported in Denmark; and bacampicillin and piperacillin were only reported in Italy.

In 2017, COP represented 46.3% (compared with 42.8% in 2009) of penicillin consumption in the community. COP consumption ranged from 7.85 DDD per 1000 inhabitants per day in Spain to 0.01 DDD per 1000 inhabitants per day in Norway. Amoxicillin/clavulanic acid was the most commonly reported COP. It represented >50% of penicillin consumption in the community in Malta (91.7%), Hungary (80.1%), Cyprus (76.6%; total care data), Italy (76.5%), Portugal (70.2%), Austria (68.5%), Croatia (68.2%), Romania (56.9%; total care data), Spain (55.2%), Greece (55.2%) and Luxembourg (53.8%), but <5% in Sweden (3.4%). Sultamicillin consumption remained <1% of penicillin consumption in the community for all countries but Estonia (2.4%), Germany (2.2%) and Lithuania (1.5%).

In 2017, PRP represented 5.0% (compared with 4.3% in 2009) of penicillin consumption in the community. PRP consumption ranged from 1.56 DDD per 1000 inhabitants per day in Denmark to 0.0001 DDD per 1000 inhabitants per day in Estonia, with no consumption reported in Bulgaria and Hungary. Flucloxacillin was the most commonly reported PRP. It represented >20% of penicillin consumption in the community in Sweden (25.2%) and the United Kingdom (23.7%) while it was not reported in Cyprus (total care data), France, Greece, Lithuania, Poland and Spain. With the exception of Lithuania, countries without flucloxacillin consumption reported consumption of cloxacillin as the sole PRP.

**Figure 2.**
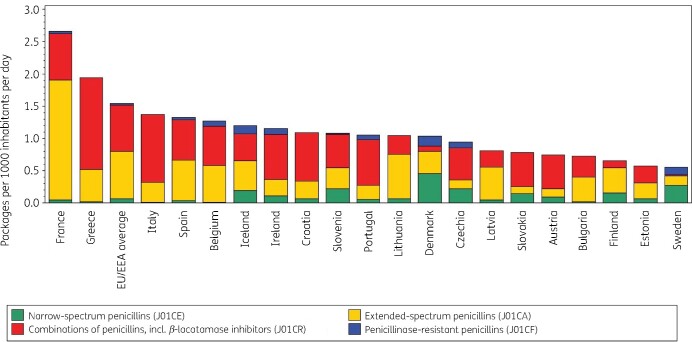
Consumption of penicillins (ATC J01C) in the community, expressed in packages per 1000 inhabitants per day, 20 EU/EEA countries, 2017. For Czechia, 2015 data are used. For Slovakia, 2016 data are used. For Cyprus and Romania, total care data, i.e. community and hospital sector combined, are used.

Figure 2 shows consumption of penicillins in the community expressed in packages per 1000 inhabitants per day for 20 EU/EEA countries in 2017. Based on this indicator, France showed the highest consumption (2.7 packages per 1000 inhabitants per day) while Sweden showed the lowest consumption (0.5 packages per 1000 inhabitants per day). Denmark shifted from rank 6 for its consumption of penicillins in DDD per 1000 inhabitants per day (among the highest prescribing countries) to rank 12 in packages per 1000 inhabitants per day (among the lowest prescribing countries), Spain moved from the 1st to the 4th rank and Sweden from the 15th to the 20th rank, while Italy moved from the 8th to the 3rd rank (Table 2). The lowest mean number of DDD per package was observed for France (4.9 DDD per package) and the highest for Spain and Sweden (10.7 DDD per package). In the EU/EEA, the number of DDD per package increased significantly over time [+0.099 (SD 0.0274) DDD per year] during 1997–2017.

**Table 2.** Ranking of consumption of penicillins (ATC J01C) in the community, expressed in DDD or packages per 1000 inhabitants per day, 20 EU/EEA countries, 2017

Country	France	Greece	Italy	Spain	Belgium	Iceland	Ireland	Croatia	Slovenia	Portugal	Lithuania	Denmark	Czechia	Latvia	Slovakia	Austria	Bulgaria	Finland	Estonia	Sweden
Ranking for packages per 1000 inhabitants per day	1	2	3	4	5	6	7	8	9	10	11	12	13	14	15	16	17	18	19	20
Ranking for DDD per 1000 inhabitants per day	2	3	8	1	4	7	5	10	11	9	12	6	13	17	14	17	16	19	20	15
Number of DDD per package	4.9	5.5	6.4	10.7	7.7	7.8	8.3	7.2	6.4	8.2	6.5	9.3	6.6	5.8	7.7	6.9	7.5	6.7	6.0	10.7

For Czechia, 2015 data are used. For Slovakia, 2016 data are used. For Cyprus and Romania, total care data are used. For Ireland, nitrofurantoin (J01XE01) consumption was not included.

### Longitudinal data analysis, 1997–2017

**Figure 3.**
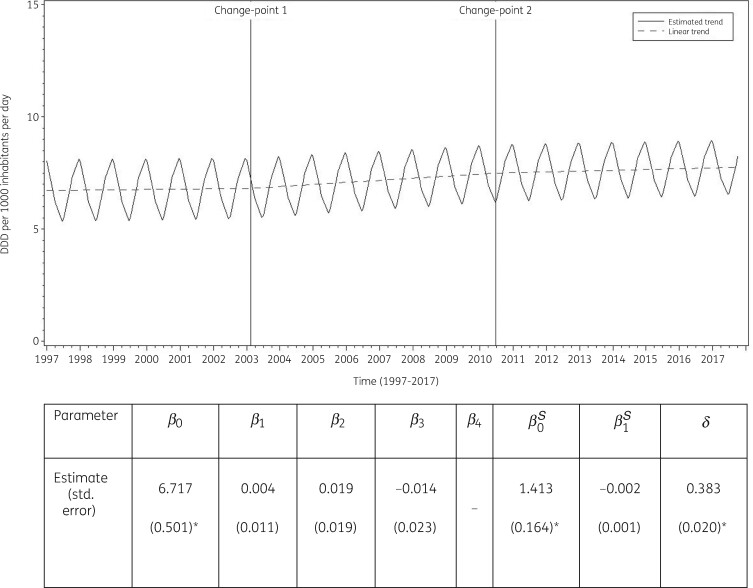
Estimated trend (solid line) and linear trend (dashed line) of consumption of penicillins (ATC J01C) in the community based on quarterly data, 25 EU/EEA countries, 1997–2017. *β_0_*, predicted consumption in the first quarter of 1997; *β_1_*, predicted increase (if positive)/decrease (if negative) in consumption per quarter; *β_2_*, predicted difference in slope after versus before the first change-point; *β_3_*, predicted difference in slope after versus before the second change-point; *β_4_*, predicted difference in slope after versus before the third change-point; *β_0_^S^*, predicted amplitude of the upward winter and downward summer peak in consumption; *β_1_^S^*, predicted increase (if positive)/decrease (if negative) of the amplitude of the upward winter and downward summer peak in consumption per quarter; *δ*, shift in timing of the upward winter and downward summer peak from one year to another. *Statistically significant at significance level 0.05.

The best fit was obtained for a model including two change-points: one in the first quarter of 2003 and another in the second quarter of 2010. The final model fits the observed data well (Figure S1). The longitudinal data analysis estimated an average consumption of penicillins in the EU/EEA of 6.717 (SE 0.501) DDD per 1000 inhabitants per day in the first quarter of 1997, which did not change significantly over time: +0.004 (SE 0.011) DDD per 1000 inhabitants per day per quarter until the first quarter of 2003; +0.022 (SE 0.019) DDD per 1000 inhabitants per day per quarter between the second quarter of 2003 and the second quarter of 2010; and +0.008 (SE 0.030) DDD per 1000 inhabitants per day per quarter afterwards. Furthermore, the longitudinal data analysis showed significant seasonal variation with an amplitude of 1.413 (SE 0.164) DDD per 1000 inhabitants per day, which did not change significantly over time: −0.002 (SE 0.001) DDD per 1000 inhabitants per day per quarter (Figure 3).

Based on the final fitted model, consumption of penicillins in the community in 1997 was significantly above average in Iceland and Slovakia, and significantly below average in Estonia, Finland, Germany, the Netherlands and the United Kingdom (observed profiles shown in Figures S2 and S3). The seasonal variation was significantly larger than average in Belgium, Italy, Lithuania, Luxembourg and Slovakia, and significantly smaller than average in Austria, Estonia, Finland, Germany, Greece, the Netherlands, Sweden and the United Kingdom. The increase in consumption of penicillins in the community between 1997 and the first quarter of 2003 was significantly larger than average in Croatia, Denmark and Poland. The increase in consumption of penicillins between the second quarter of 2003 and the second quarter of 2010 was significantly larger than average in Belgium, Italy and Luxembourg. The increase in consumption of penicillins between the third quarter of 2010 and the last quarter of 2017 was significantly larger than average in Ireland.

### Compositional data analysis, 1997–2017

The proportional consumption of ESP and COP significantly increased over time relative to that of NSP and PRP. In addition, the proportional consumption of COP significantly increased over time relative to that of ESP (Table 3).

**Table 3.** Change in the composition of the consumption of penicillins (ATC J01C) in the community, expressed in DDD (ATC/DDD index 2019) per 1000 inhabitants per day, 30 EU/EEA countries, as a function of time during 1997–2017

	NSP	ESP	COP	PRP
NSP		**−0.0586**	**−0.1183**	**−0.0295**
ESP	**0.0586**		**−0.0597**	**0.0292**
COP	**0.1183**	**0.0597**		**0.0888**
PRP	**0.0295**	**−0.0292**	**−0.0888**	

Values are estimated changes in the log ratio of the row versus column subgroup of antibiotics with increasing time. Bold type indicates a statistically significant effect; positive values represent an increase and negative values represent a decrease.

NSP, narrow-spectrum penicillins (J01CE); ESP, extended-spectrum penicillins (J01CA); COP, combinations of penicillins, including β-lactamase inhibitors (J01CR); PRP, penicillinase-resistant penicillins (J01CF).

Trends of proportional consumption of penicillins in the community for individual countries are shown in Figure S4. When comparing the composition of the consumption of penicillins (J01C) in 2017 with that in 2009, the proportion of NSP decreased for most of the participating countries. The largest decreases were observed for Denmark (−13.74%), Lithuania (−10.77%) and Hungary (−10.70%). However, increases were also observed, with the largest increases reported for Poland (+2.58%), Malta (+1.45%) and Romania (+1.42%; total care data; coverage in 2009 limited to 30%–40%). For the proportions of ESP, COP and PRP, both increases and decreases were observed between 2009 and 2017. For ESP, the largest increases were reported for Belgium (+10.12%), France (+7.80%) and Spain (+7.06%; private prescriptions included from 2016 onwards), while the largest decreases were reported for Estonia (−18.63%), Cyprus (−14.29%; total care data) and Italy (−12.64%). For COP, the largest increases were reported for Estonia (+21.84%), Croatia (+18.72%) and Hungary (+18.16%), while the largest decreases were reported for Belgium (−9.37%), Luxembourg (−8.24%) and Ireland (−7.09%). For PRP, the largest increases were reported for the United Kingdom (+4.59%), the Netherlands (+4.15%) and Denmark (+4.10%), while the largest decreases were reported for France (−2.75%) and Iceland (−2.23%).

## Discussion

Penicillins (J01C) were the most frequently consumed antibiotics in the community in the EU/EEA in 2017.^4^ Consumption of penicillins in European countries that are not part of the ESAC-Net but covered by the WHO Europe Antimicrobial Medicines Consumption Network also was substantial, ranging from 28% in Kazakhstan to 52.5% in Uzbekistan.^8^

In the EU/EEA, consumption of penicillins in the community remained high and stable between 1997 and 2017. Inter-country variability of consumption of penicillins in the community expressed in DDD per 1000 inhabitants per day was substantial, and increased when compared with data from 2009. Seasonal variation was high and remained stable over time.

Among the 46 penicillins with an ATC code, more substances were no longer prescribed in 2017 (21 out of 46) than in 2009 (12 out of 44). Overall, COP was the most frequently consumed subgroup of penicillins. Proportional consumption of COP increased in most countries at the expense of consumption of NSP or ESP. Given that total consumption of penicillins did not change significantly over time between 1997 and 2017, this implies that consumption of antibiotics from one subgroup was merely replaced by consumption of antibiotics from another subgroup, rather than being reduced overall. In Belgium, a 10% decrease in consumption of COP was accompanied by a 10% increase in consumption of ESP, most likely as the result of successful multi-faceted campaigning in the country.^9^ Country-specific consumption of COP showed great variability, ranging from 7.85 DDD per 1000 inhabitants per day in Spain to 0.01 DDD per 1000 inhabitants per day in Norway. The most frequently reported substance was amoxicillin/clavulanic acid, which represented >50% of penicillin consumption in the community in 11 countries. This finding, once again, raises concern about the appropriate prescribing of amoxicillin/clavulanic acid for respiratory tract infections (RTIs).^10^

Given that the mean number of DDD per package varied considerably and increased over time,^11^ and that antimicrobial resistance best correlates with consumption expressed in packages,^12^ we recommend evaluating antibiotic consumption expressed both in DDD per 1000 inhabitants per day and in packages per 1000 inhabitants per day.

With the exception of four penicillins that represented <1% of penicillin consumption in the community in EU/EEA countries, the penicillins used were listed in the 2019 WHO Access, Watch or Reserve (AWaRe) classification list.^13^ Most penicillins are listed as antibiotics belonging to the Access group. The antibiotics listed in the Watch group, which include pheneticillin (J01CE05), piperacillin (J01CA12), piperacillin and β-lactamase inhibitor (J01CR05) and temocillin (J01CA17), are mainly used in the hospital sector (consumption of these antibiotics in the community was <1% in the EU/EEA in 2017). Care should be taken by countries to optimize the availability of penicillins that belong to the Access group.

The continued seasonal variation in consumption of penicillins in the community found in this study confirms that penicillins are still prescribed for seasonal RTIs, which represent nearly 60% of antibiotic prescriptions in the community, even though RTIs are mostly viral in origin.^14–16^ Based on this finding, acute RTIs remain an ideal opportunity for antimicrobial stewardship activities in the community in EU/EEA countries.

For a detailed discussion on the limitations of the collected data, we refer to the article on antibacterials for systemic use, included in this series.^17^ For a discussion on the limitations of the statistical approach used in this study and potential explanations for the common change-points detected through these analyses, we refer to the tutorial included in this series.^6^

In conclusion, neither the consumption nor the seasonal variation in consumption of penicillins in the community changed over time. However, the proportional consumption of COP increased significantly over time during 1997–2017.

## Supplementary Material

dkab173_Supplementary_Data

## Supplementary data

Tables S1 and S2 and Figures S1 to S4 are available as Supplementary data at *JAC* Online.
