# Supplementary material for: Consumption of penicillins in the community, European Union/European Economic Area, 1997–2017
Source: J Antimicrob Chemother. 2021 Aug 1;76(Suppl 2):ii14–21. doi: 10.1093/jac/dkab173 (PMC8314108; doi:10.1093/jac/dkab173)
Supplement: dkab173_Supplementary_Data [file dkab173_supplementary_data.docx]

**Supplementary data**

**Table S1. Consumption of penicillins (ATC J01C) in the community, expressed in DDD (ATC/DDD index 2019) per 1000 inhabitants per day, 30 EU/EEA countries, 1997-2017.**

| **Country** | **1997** | **1998** | **1999** | **2000** | **2001** | **2002** | **2003** | **2004** | **2005** | **2006** | **2007** | **2008** | **2009** | **2010** | **2011** | **2012** | **2013** | **2014** | **2015** | **2016** | **2017** |
| --- | --- | --- | --- | --- | --- | --- | --- | --- | --- | --- | --- | --- | --- | --- | --- | --- | --- | --- | --- | --- | --- |
| **Austria** | \| - \| \| --- \| | **3.51** | **3.44** | **3.41** | **3.38** | **3.40** | **3.84** | **3.78** | **4.35** | **4.46** | **4.51** | **4.78** | **4.79** | **4.78** | **4.67** | **4.53** | **5.29** | **4.65** | **4.72** | **4.67** | **5.12** |
| NSP | \| - \| \| --- \| | 1.49 | 1.32 | 1.33 | 1.23 | 1.12 | 1.14 | 1.05 | 0.99 | 1.02 | 1.00 | 1.05 | 0.99 | 0.92 | 0.86 | 0.81 | 0.92 | 0.78 | 0.76 | 0.74 | 0.75 |
| ESP | \| - \| \| --- \| | 0.89 | 0.88 | 0.84 | 0.82 | 0.72 | 0.74 | 0.68 | 0.97 | 0.98 | 1.01 | 0.99 | 0.78 | 0.83 | 0.82 | 0.72 | 0.84 | 0.71 | 0.73 | 0.73 | 0.82 |
| COP | \| - \| \| --- \| | 1.11 | 1.23 | 1.24 | 1.32 | 1.55 | 1.95 | 2.03 | 2.39 | 2.45 | 2.49 | 2.74 | 3.00 | 3.02 | 2.98 | 2.99 | 3.52 | 3.16 | 3.22 | 3.19 | 3.54 |
| PRP | \| - \| \| --- \| | 0.01 | 0.01 | 0.01 | 0.01 | 0.01 | 0.01 | 0.01 | 0.01 | 0.01 | 0.01 | 0.01 | 0.01 | 0.01 | 0.01 | <0.01 | 0.01 | 0.01 | 0.01 | 0.01 | 0.01 |
| **Belgium** | **6.42** | **6.58** | **6.76** | **6.56** | **6.37** | **6.54** | **7.02** | **7.15** | **8.07** | **8.39** | **9.14** | **10.45** | **10.16** | **10.96** | **11.14** | **11.46** | **9.87** | **9.91** | **10.09** | **10.21** | **9.74** |
| NSP | 0.18 | 0.15 | 0.18 | 0.19 | 0.17 | 0.17 | 0.16 | 0.14 | 0.13 | 0.11 | 0.10 | 0.11 | 0.10 | 0.09 | 0.05 | 0.03 | 0.03 | 0.03 | 0.03 | 0.03 | 0.02 |
| ESP | 2.65 | 2.69 | 2.75 | 2.54 | 2.34 | 2.36 | 2.35 | 2.48 | 3.10 | 3.49 | 3.84 | 4.53 | 3.91 | 4.85 | 4.92 | 5.16 | 5.21 | 4.87 | 5.01 | 5.01 | 4.73 |
| COP | 3.29 | 3.45 | 3.55 | 3.55 | 3.60 | 3.76 | 4.26 | 4.30 | 4.62 | 4.56 | 4.98 | 5.57 | 5.90 | 5.76 | 5.91 | 6.02 | 4.37 | 4.76 | 4.80 | 4.92 | 4.74 |
| PRP | 0.30 | 0.29 | 0.28 | 0.28 | 0.27 | 0.25 | 0.24 | 0.22 | 0.23 | 0.23 | 0.22 | 0.25 | 0.26 | 0.26 | 0.26 | 0.25 | 0.26 | 0.25 | 0.25 | 0.26 | 0.25 |
| **Bulgaria** | \| - \| \| --- \| | \| - \| \| --- \| | ***7.64*** | ***8.69*** | ***10.97*** | ***8.52*** | ***6.67*** | ***7.73*** | ***5.69*** | **6.17** | **6.66** | **6.71** | **5.76** | **5.51** | **5.74** | **5.31** | **5.74** | **5.58** | **5.52** | **4.65** | **5.38** |
| NSP | - | - | *1.37* | *3.18* | *4.40* | *2.68* | *0.73* | *0.85* | *0.65* | 0.57 | 0.56 | 0.46 | 0.36 | 0.32 | 0.29 | 0.24 | 0.23 | 0.19 | 0.14 | 0.15 | 0.13 |
| ESP | - | - | *5.96* | *4.78* | *5.71* | *5.07* | *4.62* | *5.46* | *3.75* | 4.43 | 4.59 | 4.52 | 3.61 | 3.54 | 3.66 | 3.37 | 3.49 | 3.20 | 3.17 | 2.46 | 2.89 |
| COP | - | - | *0.27* | *0.63* | *0.76* | *0.73* | *1.27* | *1.39* | *1.29* | 1.17 | 1.50 | 1.73 | 1.79 | 1.65 | 1.79 | 1.70 | 2.02 | 2.19 | 2.21 | 2.04 | 2.36 |
| PRP | - | - | *0.04* | *0.10* | *0.09* | *0.04* | *0.04* | *0.02* | *<0.01* | - | - | - | - | - | - | - | - | - | - | - | - |
| **Croatia** | - | - | - | - | **6.66** | **8.82** | **9.05** | **8.74** | **8.64** | **7.64** | **8.00** | **8.00** | **7.20** | **6.65** | **6.72** | **7.73** | **7.78** | **7.95** | **8.09** | **7.88** | **7.82** |
| NSP | - | - | - | - | 1.21 | 1.82 | 1.64 | 1.74 | 1.45 | 1.48 | 1.36 | 1.29 | 1.12 | 0.91 | 0.88 | 0.82 | 0.79 | 0.72 | 0.46 | 0.55 | 0.51 |
| ESP | - | - | - | - | 2.81 | 3.39 | 3.33 | 3.49 | 3.46 | 2.97 | 2.97 | 2.71 | 2.52 | 2.04 | 1.88 | 1.83 | 1.98 | 2.03 | 2.31 | 2.11 | 1.97 |
| COP | - | - | - | - | 2.57 | 3.55 | 4.02 | 3.44 | 3.67 | 3.13 | 3.61 | 3.96 | 3.57 | 3.70 | 3.95 | 5.09 | 5.00 | 5.20 | 5.31 | 5.22 | 5.34 |
| PRP | - | - | - | - | 0.07 | 0.07 | 0.06 | 0.07 | 0.06 | 0.06 | 0.06 | 0.05 | - | - | - | <0.01 | <0.01 | <0.01 | 0.01 | <0.01 | 0.01 |
| **Cyprus** | - | - | - | - | - | - | - | - | - | ***9.73*** | ***10.62*** | ***10.29*** | ***10.76*** | ***9.69*** | ***10.33*** | ***9.29*** | ***8.70*** | ***7.82*** | ***8.95*** | ***8.74*** | ***9.33*** |
| NSP | - | - | - | - | - | - | - | - | - | *0.12* | *0.12* | *0.11* | *0.12* | *0.10* | *0.10* | *0.09* | *0.07* | *0.07* | *0.08* | *0.08* | *0.07* |
| ESP | - | - | - | - | - | - | - | - | - | *4.46* | *4.70* | *4.01* | *3.86* | *3.24* | *3.37* | *3.09* | *2.63* | *2.15* | *2.35* | *2.10* | *2.02* |
| COP | - | - | - | - | - | - | - | - | - | *5.12* | *5.78* | *6.14* | *6.75* | *6.33* | *6.84* | *6.09* | *5.99* | *5.59* | *6.51* | *6.54* | *7.22* |
| PRP | - | - | - | - | - | - | - | - | - | *0.03* | *0.03* | *0.02* | *0.03* | *0.03* | *0.02* | *0.02* | *0.02* | *0.02* | *0.02* | *0.02* | *0.03* |
| **Czechia** | - | **6.45** | **6.48** | - | - | - | **5.41** | **5.23** | **5.47** | **4.99** | **5.27** | **5.71** | **5.95** | **5.76** | **6.16** | **5.21** | **6.05** | **6.08** | **6.26** | - | - |
| NSP | - | 2.68 | 2.53 | - | - | - | 2.25 | 1.92 | 1.89 | 1.99 | 2.04 | 2.02 | 2.06 | 1.99 | 2.12 | 1.48 | 1.89 | 1.89 | 1.89 | - | - |
| ESP | - | 2.37 | 2.50 | - | - | - | 1.74 | 1.47 | 1.68 | 1.33 | 1.33 | 1.06 | 1.18 | 1.12 | 1.28 | 1.16 | 1.34 | 1.17 | 1.18 | - | - |
| COP | - | 1.26 | 1.32 | - | - | - | 1.33 | 1.75 | 1.90 | 1.66 | 1.90 | 2.60 | 2.68 | 2.61 | 2.72 | 2.54 | 2.79 | 2.97 | 3.14 | - | - |
| PRP | - | 0.14 | 0.12 | - | - | - | 0.10 | 0.09 | <0.01 | <0.01 | \| - \| \| --- \| | 0.03 | 0.04 | 0.04 | 0.04 | 0.03 | 0.03 | 0.05 | 0.05 | - | - |
| Country, community consumption of penicillins (J01C); NSP, consumption of narrow-spectrum penicillins (J01CE); ESP, consumption of extended-spectrum penicillins (J01CA); COP, consumption of combination of penicillins, including β-lactamase inhibitors (J01CR); PRP, consumption of penicillinase-resistant penicillins (J01CF); **-**, no consumption reported; Numbers reported in *italic* are total care data, i.e. community and hospital sector combined; ^a^Data for Romania have a coverage in 2009 limited to 30-40%; ^b^Data for Spain include private prescriptions from 2016 onwards. | | | | | | | | | | | | | | | | | | | | | |
| **Denmark** | **6.90** | **7.20** | **6.90** | **7.23** | **7.65** | **7.92** | **8.11** | **8.43** | **8.66** | **9.03** | **9.64** | **9.34** | **9.30** | **9.66** | **10.13** | **9.53** | **9.79** | **9.72** | **9.87** | **9.79** | **9.52** |
| NSP | 4.56 | 4.80 | 4.47 | 4.74 | 4.90 | 4.99 | 5.07 | 5.21 | 5.26 | 5.38 | 5.63 | 5.18 | 5.00 | 5.13 | 5.22 | 4.58 | 4.56 | 4.29 | 4.26 | 4.07 | 3.81 |
| ESP | 1.98 | 1.98 | 1.93 | 1.95 | 2.08 | 2.13 | 2.15 | 2.25 | 2.38 | 2.53 | 2.79 | 2.85 | 2.88 | 2.90 | 3.10 | 3.03 | 3.12 | 3.20 | 3.28 | 3.31 | 3.36 |
| COP | 0.02 | 0.02 | 0.02 | 0.02 | 0.02 | 0.03 | 0.03 | 0.04 | 0.05 | 0.08 | 0.12 | 0.18 | 0.27 | 0.45 | 0.59 | 0.70 | 0.81 | 0.87 | 0.95 | 0.94 | 0.79 |
| PRP | 0.34 | 0.40 | 0.48 | 0.53 | 0.65 | 0.77 | 0.85 | 0.92 | 0.97 | 1.04 | 1.09 | 1.13 | 1.14 | 1.17 | 1.22 | 1.21 | 1.30 | 1.36 | 1.38 | 1.47 | 1.56 |
| **Estonia** | - | - | - | - | ***5.49*** | **3.19** | **3.09** | **2.92** | **3.19** | **3.15** | **3.41** | **3.31** | **3.03** | **2.94** | **3.18** | **3.08** | **3.09** | **3.18** | **3.28** | **3.37** | **3.47** |
| NSP | - | - | - | - | *0.41* | 0.30 | 0.23 | 0.32 | 0.40 | 0.29 | 0.31 | 0.31 | 0.24 | 0.25 | 0.24 | 0.22 | 0.21 | 0.20 | 0.14 | 0.17 | 0.17 |
| ESP | - | - | - | - | *4.51* | 2.63 | 2.45 | 2.13 | 2.20 | 2.20 | 2.28 | 2.12 | 1.96 | 1.82 | 1.91 | 1.80 | 1.68 | 1.71 | 1.71 | 1.66 | 1.60 |
| COP | - | - | - | - | *0.52* | 0.26 | 0.42 | 0.48 | 0.59 | 0.65 | 0.82 | 0.87 | 0.83 | 0.87 | 1.03 | 1.07 | 1.20 | 1.28 | 1.43 | 1.53 | 1.70 |
| PRP | - | - | - | - | *0.05* | \| - \| \| --- \| | <0.01 | <0.01 | <0.01 | <0.01 | <0.01 | 0.01 | <0.01 | <0.01 | <0.01 | <0.01 | <0.01 | <0.01 | <0.01 | <0.01 | <0.01 |
| **Finland** | **4.92** | **4.58** | **4.42** | **4.59** | **4.92** | **4.07** | **4.60** | **4.14** | **4.53** | **4.37** | **4.77** | **4.75** | **4.75** | **5.12** | **5.12** | **5.00** | **4.82** | **4.95** | **4.86** | **4.76** | **4.40** |
| NSP | 2.52 | 2.41 | 2.23 | 2.14 | 2.10 | 1.54 | 1.78 | 1.57 | 1.59 | 1.57 | 1.62 | 1.38 | 1.38 | 1.43 | 1.36 | 1.32 | 1.26 | 1.25 | 1.23 | 1.19 | 1.12 |
| ESP | 2.20 | 1.95 | 1.94 | 2.13 | 2.29 | 2.00 | 2.19 | 1.97 | 2.25 | 2.15 | 2.36 | 2.53 | 2.53 | 2.78 | 2.80 | 2.70 | 2.65 | 2.73 | 2.69 | 2.70 | 2.49 |
| COP | 0.13 | 0.16 | 0.20 | 0.27 | 0.47 | 0.47 | 0.58 | 0.55 | 0.64 | 0.61 | 0.75 | 0.82 | 0.82 | 0.91 | 0.97 | 0.96 | 0.88 | 0.93 | 0.90 | 0.82 | 0.73 |
| PRP | 0.06 | 0.06 | 0.06 | 0.06 | 0.05 | 0.05 | 0.05 | 0.05 | 0.04 | 0.04 | 0.04 | 0.02 | 0.02 | <0.01 | <0.01 | 0.03 | 0.04 | 0.05 | 0.05 | 0.05 | 0.06 |
| **France** | **12.00** | **12.24** | **12.18** | **11.29** | **11.30** | **11.31** | **9.60** | **8.83** | **9.84** | **9.95** | **10.17** | **10.03** | **10.91** | **10.60** | **11.17** | **11.75** | **12.42** | **12.17** | **12.68** | **13.31** | **13.00** |
| NSP | 0.33 | 0.29 | 0.24 | 0.21 | 0.19 | 0.28 | 0.16 | 0.17 | 0.15 | 0.15 | 0.15 | 0.14 | 0.15 | 0.15 | 0.15 | 0.15 | 0.17 | 0.17 | 0.16 | 0.18 | 0.17 |
| ESP | 8.52 | 8.72 | 8.51 | 7.44 | 6.90 | 6.26 | 5.35 | 4.74 | 5.19 | 5.33 | 5.42 | 5.47 | 6.00 | 5.63 | 6.10 | 6.46 | 7.10 | 6.95 | 7.50 | 8.12 | 8.17 |
| COP | 2.59 | 2.72 | 2.85 | 3.10 | 3.67 | 4.27 | 3.63 | 3.46 | 4.05 | 4.04 | 4.18 | 3.99 | 4.33 | 4.40 | 4.59 | 4.86 | 4.89 | 4.79 | 4.80 | 4.83 | 4.51 |
| PRP | 0.56 | 0.51 | 0.58 | 0.54 | 0.54 | 0.49 | 0.46 | 0.47 | 0.46 | 0.44 | 0.43 | 0.43 | 0.43 | 0.42 | 0.32 | 0.28 | 0.26 | 0.26 | 0.21 | 0.18 | 0.15 |
| **Germany** | **3.45** | **3.66** | **3.66** | **3.88** | **3.39** | **3.43** | **3.51** | **3.10** | **3.32** | **3.24** | **3.33** | **3.27** | **3.16** | **3.03** | **2.87** | **3.30** | **3.53** | **3.36** | **3.30** | **3.42** | **3.57** |
| NSP | 1.81 | 1.89 | 1.83 | 1.94 | 1.53 | 1.47 | 1.44 | 1.18 | 1.10 | 1.09 | 1.00 | 0.94 | 0.85 | 0.81 | 0.70 | 0.85 | 0.84 | 0.80 | 0.71 | 0.74 | 0.69 |
| ESP | 1.54 | 1.66 | 1.71 | 1.79 | 1.70 | 1.79 | 1.93 | 1.77 | 2.02 | 1.96 | 2.13 | 2.11 | 2.08 | 1.98 | 1.92 | 2.16 | 2.36 | 2.22 | 2.23 | 2.29 | 2.45 |
| COP | 0.08 | 0.10 | 0.11 | 0.13 | 0.15 | 0.16 | 0.13 | 0.14 | 0.17 | 0.17 | 0.19 | 0.20 | 0.22 | 0.23 | 0.25 | 0.29 | 0.32 | 0.34 | 0.36 | 0.38 | 0.41 |
| PRP | 0.02 | 0.02 | 0.02 | 0.02 | 0.02 | 0.02 | 0.02 | 0.02 | 0.02 | 0.01 | 0.01 | 0.01 | 0.01 | 0.01 | 0.01 | 0.01 | 0.01 | 0.01 | 0.01 | 0.01 | 0.01 |
| **Greece** | **6.59** | **5.54** | **7.10** | **7.07** | **7.02** | **6.83** | **7.04** | ***7.16*** | ***7.67*** | ***9.63*** | ***8.99*** | ***10.17*** | **8.87** | ***8.74*** | **8.34** | **8.62** | **8.45** | **8.01** | **9.78** | **10.88** | **10.76** |
| NSP | 0.53 | 0.53 | 0.53 | 0.53 | 0.50 | 0.37 | 0.26 | *0.25* | *0.26* | *2.16* | *1.89* | *1.65* | 0.72 | *0.07* | 0.12 | 0.03 | 0.01 | 0.03 | 0.04 | 0.22 | 0.19 |
| ESP | 4.45 | 3.22 | 4.10 | 3.79 | 3.85 | 3.52 | 3.60 | *3.34* | *3.78* | *3.99* | *3.87* | *4.89* | 4.61 | *4.24* | 4.25 | 4.49 | 4.52 | 3.15 | 4.59 | 5.23 | 4.50 |
| COP | 1.59 | 1.77 | 2.46 | 2.75 | 2.67 | 2.94 | 3.18 | *3.56* | *3.62* | *3.48* | *3.23* | *3.63* | 3.54 | *4.42* | 3.96 | 4.10 | 3.92 | 4.84 | 5.15 | 5.40 | 6.08 |
| PRP | 0.02 | 0.02 | 0.01 | <0.01 | <0.01 | <0.01 | <0.01 | *0.01* | *0.01* | *0.01* | *<0.01* | *<0.01* | <0.01 | *0.01* | <0.01 | <0.01 | <0.01 | <0.01 | <0.01 | 0.03 | <0.01 |
| Country, community consumption of penicillins (J01C); NSP, consumption of narrow-spectrum penicillins (J01CE); ESP, consumption of extended-spectrum penicillins (J01CA); COP, consumption of combination of penicillins, including β-lactamase inhibitors (J01CR); PRP, consumption of penicillinase-resistant penicillins (J01CF); **-**, no consumption reported; Numbers reported in *italic* are total care data, i.e. community and hospital sector combined; ^a^Data for Romania have a coverage in 2009 limited to 30-40%; ^b^Data for Spain include private prescriptions from 2016 onwards. | | | | | | | | | | | | | | | | | | | | | |
|  |  |  |  |  |  |  |  |  |  |  |  |  |  |  |  |  |  |  |  |  |  |
|  |  |  |  |  |  |  |  |  |  |  |  |  |  |  |  |  |  |  |  |  |  |
|  |  |  |  |  |  |  |  |  |  |  |  |  |  |  |  |  |  |  |  |  |  |
| **Hungary** | - | **6.32** | **7.88** | **6.22** | **6.38** | **5.86** | **6.75** | **6.16** | **6.22** | **5.14** | **4.96** | **4.36** | **4.94** | **4.65** | **4.87** | **4.59** | **4.65** | **4.63** | **4.78** | **4.39** | **4.51** |
| NSP | - | 1.15 | 1.34 | 1.10 | 1.09 | 0.97 | 1.31 | 1.09 | 0.81 | 0.83 | 0.84 | 0.73 | 0.67 | 0.54 | 0.45 | 0.39 | 0.35 | 0.26 | 0.12 | 0.13 | 0.13 |
| ESP | - | 2.62 | 3.18 | 2.37 | 2.45 | 2.17 | 2.39 | 2.04 | 1.92 | 1.22 | 1.34 | 1.23 | 1.19 | 1.05 | 1.13 | 1.06 | 1.04 | 0.81 | 0.85 | 0.79 | 0.75 |
| COP | - | 2.55 | 3.36 | 2.75 | 2.85 | 2.71 | 3.05 | 3.03 | 3.49 | 3.10 | 2.78 | 2.39 | 3.08 | 3.06 | 3.28 | 3.14 | 3.26 | 3.57 | 3.81 | 3.47 | 3.62 |
| PRP | - | - | <0.01 | <0.01 | <0.01 | <0.01 | <0.01 | <0.01 | - | - | - | - | - | - | - | - | - | - | - | - | - |
| **Iceland** | ***10.59*** | ***11.20*** | ***10.40*** | ***10.48*** | ***10.12*** | ***10.69*** | ***9.68*** | ***8.99*** | ***9.63*** | **8.31** | **7.71** | **8.66** | **8.22** | ***9.60*** | ***9.65*** | ***9.61*** | ***9.23*** | **8.23** | **8.34** | **9.07** | **9.34** |
| NSP | *3.81* | *3.97* | *3.25* | *3.08* | *2.92* | *2.92* | *2.52* | *2.93* | *3.05* | 2.46 | 1.95 | 2.67 | 2.35 | *2.51* | *2.50* | *2.44* | *2.15* | 2.09 | 1.90 | 1.97 | 1.88 |
| ESP | *4.33* | *4.60* | *4.35* | *4.21* | *3.95* | *4.19* | *4.03* | *2.87* | *3.05* | 2.72 | 2.63 | 2.61 | 2.56 | *3.14* | *3.14* | *3.29* | *2.96* | 2.52 | 2.65 | 3.12 | 3.54 |
| COP | *1.19* | *1.33* | *1.40* | *1.85* | *1.98* | *2.31* | *1.78* | *1.85* | *2.17* | 2.08 | 2.07 | 2.35 | 2.36 | *2.67* | *2.67* | *2.61* | *2.91* | 2.74 | 2.93 | 3.09 | 3.04 |
| PRP | *1.26* | *1.30* | *1.39* | *1.34* | *1.28* | *1.28* | *1.35* | *1.34* | *1.37* | 1.05 | 1.06 | 1.03 | 0.95 | *1.28* | *1.34* | *1.26* | *1.20* | 0.89 | 0.86 | 0.89 | 0.87 |
| **Ireland** | - | **5.96** | **6.67** | **6.54** | **6.80** | **6.66** | **7.32** | **7.16** | **7.45** | **7.65** | **8.39** | **8.25** | **7.74** | **7.76** | **8.83** | **9.03** | **9.46** | **9.67** | **11.34** | **10.25** | **9.54** |
| NSP | - | 0.78 | 0.81 | 0.82 | 0.77 | 0.72 | 0.76 | 0.83 | 0.81 | 0.86 | 0.96 | 0.90 | 0.85 | 0.87 | 0.98 | 1.00 | 1.06 | 1.04 | 1.05 | 1.09 | 1.16 |
| ESP | - | 2.90 | 3.10 | 2.78 | 2.72 | 2.44 | 2.70 | 2.35 | 2.41 | 2.43 | 2.57 | 2.42 | 2.24 | 2.17 | 2.38 | 2.44 | 2.59 | 2.82 | 3.56 | 3.08 | 3.20 |
| COP | - | 1.60 | 2.04 | 2.15 | 2.52 | 2.68 | 3.01 | 3.11 | 3.30 | 3.41 | 3.79 | 3.91 | 3.67 | 3.66 | 4.42 | 4.54 | 4.62 | 3.98 | 4.49 | 4.56 | 3.85 |
| PRP | - | 0.68 | 0.72 | 0.80 | 0.80 | 0.82 | 0.85 | 0.87 | 0.93 | 0.95 | 1.07 | 1.02 | 0.98 | 1.06 | 1.05 | 1.06 | 1.19 | 1.83 | 2.25 | 1.52 | 1.33 |
| **Italy** | - | - | **7.48** | **7.43** | **8.12** | **7.81** | **8.52** | **8.36** | **8.90** | **9.29** | **9.90** | **10.28** | **10.25** | **1<0.01** | **10.49** | **10.32** | **10.79** | **10.49** | **10.38** | **10.30** | **8.79** |
| NSP | - | - | 0.06 | 0.05 | 0.04 | 0.03 | 0.01 | 0.01 | 0.01 | 0.01 | <0.01 | <0.01 | <0.01 | <0.01 | <0.01 | <0.01 | <0.01 | <0.01 | <0.01 | <0.01 | <0.01 |
| ESP | - | - | 4.74 | 4.55 | 4.90 | 4.54 | 4.63 | 4.38 | 4.49 | 4.37 | 4.28 | 4.13 | 3.68 | 3.38 | 3.17 | 3.01 | 2.93 | 2.63 | 2.52 | 2.45 | 2.05 |
| COP | - | - | 2.67 | 2.80 | 3.14 | 3.21 | 3.86 | 3.96 | 4.39 | 4.89 | 5.60 | 6.13 | 6.55 | 6.60 | 7.32 | 7.31 | 7.85 | 7.86 | 7.86 | 7.83 | 6.73 |
| PRP | - | - | 0.01 | 0.03 | 0.04 | 0.03 | 0.02 | 0.02 | 0.02 | 0.01 | 0.01 | 0.01 | 0.01 | 0.01 | 0.01 | 0.01 | 0.01 | 0.01 | 0.01 | 0.01 | 0.01 |
| **Latvia** | - | - | - | - | - | **3.55** | - | **3.91** | **3.83** | **3.86** | **4.19** | **3.54** | **3.43** | **3.70** | **4.08** | **4.17** | **4.40** | **4.09** | **4.37** | **4.31** | **4.66** |
| NSP | - | - | - | - | - | 0.15 | - | 0.19 | 0.18 | 0.16 | 0.14 | 0.14 | 0.16 | 0.05 | <0.01 | 0.01 | 0.04 | 0.07 | 0.07 | 0.06 | 0.08 |
| ESP | - | - | - | - | - | 2.77 | - | 2.91 | 2.65 | 2.66 | 2.65 | 2.32 | 2.36 | 2.70 | 3.05 | 3.02 | 3.11 | 2.81 | 2.96 | 2.87 | 3.03 |
| COP | - | - | - | - | - | 0.62 | - | 0.81 | 0.99 | 1.04 | 1.39 | 1.08 | 0.91 | 0.95 | 1.03 | 1.13 | 1.24 | 1.21 | 1.34 | 1.38 | 1.55 |
| PRP | - | - | - | - | - | 0.01 | - | 0.01 | <0.01 | \| - \| \| --- \| | <0.01 | \| - \| \| --- \| | <0.01 | <0.01 | \| - \| \| --- \| | <0.01 | 0.01 | <0.01 | <0.01 | <0.01 | <0.01 |
| **Lithuania** | - | - | - | - | - | - | - | - | - | ***14.50*** | ***9.51*** | ***8.92*** | ***6.81*** | ***6.38*** | ***6.91*** | **6.20** | **7.22** | **6.09** | **6.51** | **6.52** | **6.73** |
| NSP | - | - | - | - | - | - | - | - | - | *7.06* | *3.31* | *2.32* | *0.89* | *0.51* | *0.43* | 0.18 | 0.18 | 0.16 | 0.13 | 0.14 | 0.15 |
| ESP | - | - | - | - | - | - | - | - | - | *5.92* | *4.88* | *5.34* | *4.71* | *4.71* | *5.26* | 4.81 | 5.56 | 4.67 | 4.96 | 4.84 | 4.87 |
| COP | - | - | - | - | - | - | - | - | - | *0.74* | *1.32* | *1.24* | *1.18* | *1.13* | *1.22* | 1.21 | 1.49 | 1.26 | 1.42 | 1.54 | 1.70 |
| PRP | - | - | - | - | - | - | - | - | - | *0.78* | *<0.01* | *0.01* | *0.03* | *0.03* | *-* | - | - | - | - | <0.01 | <0.01 |
| Country, community consumption of penicillins (J01C); NSP, consumption of narrow-spectrum penicillins (J01CE); ESP, consumption of extended-spectrum penicillins (J01CA); COP, consumption of combination of penicillins, including β-lactamase inhibitors (J01CR); PRP, consumption of penicillinase-resistant penicillins (J01CF); **-**, no consumption reported; Numbers reported in *italic* are total care data, i.e. community and hospital sector combined; ^a^Data for Romania have a coverage in 2009 limited to 30-40%; ^b^Data for Spain include private prescriptions from 2016 onwards. | | | | | | | | | | | | | | | | | | | | | |
|  |  |  |  |  |  |  |  |  |  |  |  |  |  |  |  |  |  |  |  |  |  |
|  |  |  |  |  |  |  |  |  |  |  |  |  |  |  |  |  |  |  |  |  |  |
|  |  |  |  |  |  |  |  |  |  |  |  |  |  |  |  |  |  |  |  |  |  |
| **Luxembourg** | **6.36** | **7.93** | **6.73** | **6.57** | **7.03** | **7.39** | **8.09** | **7.34** | **7.92** | **7.78** | **8.52** | **8.56** | **9.06** | **9.03** | **9.04** | **9.21** | **9.24** | **8.64** | **9.02** | **8.42** | **6.79** |
| NSP | 0.19 | 0.17 | 0.16 | 0.18 | 0.15 | 0.14 | 0.16 | 0.17 | 0.17 | 0.14 | 0.14 | 0.12 | 0.09 | 0.08 | 0.05 | 0.03 | 0.02 | 0.01 | 0.01 | 0.01 | 0.02 |
| ESP | 3.14 | 4.65 | 3.16 | 3.06 | 3.10 | 3.09 | 3.14 | 2.64 | 2.75 | 2.60 | 2.90 | 2.97 | 3.16 | 3.11 | 3.02 | 3.01 | 3.07 | 2.86 | 3.28 | 3.46 | 2.73 |
| COP | 2.81 | 2.90 | 3.21 | 3.14 | 3.59 | 3.98 | 4.61 | 4.36 | 4.82 | 4.85 | 5.31 | 5.29 | 5.62 | 5.66 | 5.81 | 6.02 | 5.99 | 5.61 | 5.58 | 4.82 | 3.65 |
| PRP | 0.22 | 0.21 | 0.20 | 0.20 | 0.20 | 0.19 | 0.17 | 0.18 | 0.19 | 0.19 | 0.18 | 0.18 | 0.19 | 0.18 | 0.16 | 0.16 | 0.16 | 0.15 | 0.14 | 0.14 | 0.40 |
| **Malta** | - | - | - | - | - | - | - | - | - | - | **5.89** | **6.06** | **6.10** | **6.56** | **6.81** | **6.01** | **6.36** | **6.45** | **5.77** | **5.93** | **7.34** |
| NSP | - | - | - | - | - | - | - | - | - | - | 0.08 | 0.02 | 0.03 | 0.05 | 0.07 | 0.05 | 0.06 | 0.05 | 0.11 | 0.12 | 0.14 |
| ESP | - | - | - | - | - | - | - | - | - | - | 0.93 | 0.84 | 0.75 | 0.73 | 0.72 | 0.62 | 0.64 | 0.44 | 0.33 | 0.30 | 0.37 |
| COP | - | - | - | - | - | - | - | - | - | - | 4.79 | 5.13 | 5.25 | 5.76 | 5.98 | 5.34 | 5.65 | 5.96 | 5.33 | 5.28 | 6.74 |
| PRP | - | - | - | - | - | - | - | - | - | - | 0.09 | 0.07 | 0.07 | 0.03 | 0.03 | 0.01 | <0.01 | 0.01 | 0.01 | 0.24 | 0.10 |
| **Netherlands** | **2.85** | **2.80** | **2.82** | **2.78** | **2.79** | **2.78** | **2.81** | **2.75** | **2.97** | **3.16** | **3.15** | **3.21** | **3.24** | **3.16** | **3.23** | **3.25** | **3.17** | **3.07** | **3.12** | **3.09** | **2.92** |
| NSP | 0.56 | 0.53 | 0.52 | 0.52 | 0.49 | 0.45 | 0.44 | 0.42 | 0.44 | 0.50 | 0.46 | 0.41 | 0.39 | 0.37 | 0.35 | 0.33 | 0.31 | 0.30 | 0.23 | 0.24 | 0.22 |
| ESP | 1.45 | 1.42 | 1.37 | 1.25 | 1.21 | 1.18 | 1.18 | 1.13 | 1.24 | 1.26 | 1.26 | 1.26 | 1.26 | 1.20 | 1.27 | 1.29 | 1.33 | 1.29 | 1.42 | 1.38 | 1.29 |
| COP | 0.61 | 0.63 | 0.69 | 0.77 | 0.83 | 0.89 | 0.92 | 0.92 | 1.00 | 1.07 | 1.10 | 1.18 | 1.21 | 1.20 | 1.21 | 1.21 | 1.11 | 1.03 | 1.04 | 1.01 | 0.95 |
| PRP | 0.23 | 0.22 | 0.23 | 0.24 | 0.25 | 0.25 | 0.27 | 0.28 | 0.29 | 0.32 | 0.32 | 0.36 | 0.38 | 0.38 | 0.39 | 0.41 | 0.41 | 0.44 | 0.43 | 0.46 | 0.46 |
| **Norway** | - | **6.41** | - | - | **6.34** | **6.29** | **6.29** | **6.26** | **6.66** | **5.98** | **6.28** | **6.42** | **6.25** | **6.41** | **6.48** | **6.44** | **6.17** | **6.09** | **6.08** | **5.84** | **5.61** |
| NSP | - | 4.77 | - | - | 4.38 | 4.15 | 4.04 | 3.90 | 4.22 | 3.75 | 3.85 | 3.86 | 3.65 | 3.68 | 3.70 | 3.59 | 3.38 | 3.20 | 3.24 | 3.10 | 3.00 |
| ESP | - | 1.46 | - | - | 1.68 | 1.76 | 1.79 | 1.86 | 2.00 | 1.76 | 1.90 | 2.00 | 2.02 | 2.12 | 2.13 | 2.19 | 2.24 | 2.26 | 2.19 | 2.09 | 1.98 |
| COP | - | 0.01 | - | - | 0.01 | 0.01 | 0.01 | <0.01 | \| - \| \| --- \| | <0.01 | <0.01 | <0.01 | <0.01 | <0.01 | <0.01 | <0.01 | <0.01 | 0.01 | 0.01 | 0.01 | 0.01 |
| PRP | - | 0.17 | - | - | 0.28 | 0.37 | 0.45 | 0.50 | 0.44 | 0.47 | 0.53 | 0.56 | 0.58 | 0.61 | 0.65 | 0.67 | 0.55 | 0.62 | 0.64 | 0.63 | 0.61 |
| **Poland** | - | **6.30** | **7.07** | **6.88** | **7.94** | **6.88** | - | **4.91** | **5.16** | - | **7.06** | **6.82** | **7.17** | **6.36** | **7.92** | **6.18** | **6.37** | **6.01** | **7.06** | **6.82** | **6.63** |
| NSP | - | 1.39 | 1.59 | 0.59 | 1.08 | 0.48 | - | 0.29 | 0.30 | - | 0.17 | 0.16 | 0.15 | 0.14 | 0.11 | 0.15 | 0.16 | 0.21 | 0.34 | 0.32 | 0.31 |
| ESP | - | 3.81 | 4.47 | 5.13 | 5.37 | 4.92 | - | 4.20 | 4.44 | - | 3.97 | 3.66 | 3.73 | 3.22 | 3.34 | 3.44 | 3.61 | 3.27 | 3.74 | 3.54 | 3.48 |
| COP | - | 0.98 | 0.92 | 1.10 | 1.45 | 1.46 | - | 0.41 | 0.41 | - | 2.91 | 3.01 | 3.29 | 2.99 | 4.47 | 2.58 | 2.60 | 2.52 | 2.97 | 2.96 | 2.84 |
| PRP | - | 0.12 | 0.09 | 0.06 | 0.04 | 0.03 | - | 0.01 | 0.01 | - | <0.01 | 0.01 | 0.01 | 0.01 | <0.01 | <0.01 | 0.01 | 0.01 | 0.01 | 0.01 | <0.01 |
| **Portugal** | **7.06** | **7.35** | **8.05** | **8.21** | **8.04** | **8.95** | **8.13** | **7.73** | **8.21** | **7.93** | - | **7.95** | **8.19** | **8.25** | **8.40** | **8.43** | **7.55** | **7.89** | **8.28** | **8.62** | **8.67** |
| NSP | 0.05 | 0.05 | 0.05 | 0.05 | 0.04 | 0.10 | 0.01 | 0.09 | 0.02 | 0.01 | - | 0.02 | 0.02 | 0.04 | 0.03 | 0.03 | 0.01 | 0.01 | 0.01 | 0.01 | 0.01 |
| ESP | 3.71 | 3.44 | 3.43 | 3.13 | 2.77 | 2.51 | 2.32 | 2.09 | 2.14 | 2.01 | - | 1.66 | 1.62 | 1.55 | 1.69 | 1.69 | 1.39 | 1.50 | 1.64 | 1.79 | 1.80 |
| COP | 2.63 | 3.16 | 3.88 | 4.35 | 4.58 | 5.69 | 5.10 | 4.88 | 5.44 | 5.30 | - | 5.72 | 6.00 | 6.12 | 6.18 | 6.21 | 5.71 | 5.94 | 6.19 | 6.38 | 6.08 |
| PRP | 0.67 | 0.69 | 0.69 | 0.68 | 0.66 | 0.64 | 0.70 | 0.67 | 0.62 | 0.61 | - | 0.55 | 0.55 | 0.54 | 0.49 | 0.51 | 0.45 | 0.44 | 0.44 | 0.43 | 0.77 |
| Country, community consumption of penicillins (J01C); NSP, consumption of narrow-spectrum penicillins (J01CE); ESP, consumption of extended-spectrum penicillins (J01CA); COP, consumption of combination of penicillins, including β-lactamase inhibitors (J01CR); PRP, consumption of penicillinase-resistant penicillins (J01CF); **-**, no consumption reported; Numbers reported in *italic* are total care data, i.e. community and hospital sector combined; ^a^Data for Romania have a coverage in 2009 limited to 30-40%; ^b^Data for Spain include private prescriptions from 2016 onwards. | | | | | | | | | | | | | | | | | | | | | |
|  |  |  |  |  |  |  |  |  |  |  |  |  |  |  |  |  |  |  |  |  |  |
|  |  |  |  |  |  |  |  |  |  |  |  |  |  |  |  |  |  |  |  |  |  |
|  |  |  |  |  |  |  |  |  |  |  |  |  |  |  |  |  |  |  |  |  |  |
| **Romania^a^** | - | - | - | - | - | - | - | - | - | - | - | - | **3.77** | - | ***13.22*** | ***12.77*** | ***13.07*** | ***12.08*** | ***13.54*** | ***11.49*** | ***11.22*** |
| NSP | - | - | - | - | - | - | - | - | - | - | - | - | 0.16 | - | *1.19* | *1.00* | *0.91* | *0.79* | *0.87* | *0.67* | *0.63* |
| ESP | - | - | - | - | - | - | - | - | - | - | - | - | 1.07 | - | *6.16* | *5.63* | *5.34* | *5.35* | *5.19* | *3.86* | *3.65* |
| COP | - | - | - | - | - | - | - | - | - | - | - | - | 2.40 | - | *4.88* | *5.24* | *6.00* | *5.21* | *6.77* | *6.44* | *6.48* |
| PRP | - | - | - | - | - | - | - | - | - | - | - | - | 0.14 | - | *0.98* | *0.90* | *0.81* | *0.73* | *0.71* | *0.52* | *0.47* |
| **Slovakia** | - | - | **12.29** | **13.25** | **13.51** | **11.83** | **12.44** | **10.05** | **10.26** | **7.70** | **7.87** | **6.98** | **7.05** | - | ***6.90*** | **5.76** | **6.53** | **6.17** | **6.42** | **6.02** | - |
| NSP | - | - | 4.40 | 6.02 | 6.25 | 5.50 | 5.55 | 4.59 | 4.45 | 2.41 | 2.29 | 1.89 | 1.84 | - | *1.48* | 1.31 | 1.40 | 1.25 | 1.26 | 1.22 | - |
| ESP | - | - | 5.38 | 4.57 | 4.23 | 3.19 | 3.55 | 3.02 | 2.96 | 2.35 | 2.11 | 1.68 | 1.55 | - | *1.31* | 1.21 | 1.39 | 1.02 | 1.04 | 0.93 | - |
| COP | - | - | 2.48 | 2.60 | 2.96 | 3.07 | 3.28 | 2.40 | 2.85 | 2.94 | 3.48 | 3.42 | 3.66 | - | *4.11* | 3.25 | 3.74 | 3.90 | 4.13 | 3.87 | - |
| PRP | - | - | 0.03 | 0.06 | 0.07 | 0.07 | 0.06 | 0.04 | <0.01 | <0.01 | <0.01 | <0.01 | <0.01 | - | *<0.01* | - | - | - | - | - | - |
| **Slovenia** | **7.75** | **8.54** | **8.64** | **7.78** | **7.73** | **7.16** | **7.63** | **7.47** | **6.96** | **6.65** | **7.41** | **6.96** | **7.04** | **7.11** | **7.13** | **7.12** | **7.17** | **6.93** | **6.97** | **6.81** | **6.87** |
| NSP | 2.88 | 2.92 | 2.57 | 2.45 | 2.62 | 2.34 | 2.59 | 2.50 | 1.90 | 1.97 | 2.18 | 2.08 | 1.95 | 1.83 | 1.91 | 1.95 | 1.82 | 1.62 | 1.51 | 1.66 | 1.57 |
| ESP | 2.27 | 2.17 | 2.08 | 2.04 | 2.06 | 1.84 | 2.14 | 2.12 | 2.15 | 2.06 | 2.32 | 2.13 | 2.23 | 2.36 | 2.27 | 2.21 | 2.32 | 2.23 | 2.30 | 2.16 | 2.27 |
| COP | 2.48 | 3.34 | 3.88 | 3.17 | 2.92 | 2.84 | 2.74 | 2.69 | 2.74 | 2.44 | 2.77 | 2.72 | 2.71 | 2.76 | 2.78 | 2.80 | 2.89 | 2.91 | 2.98 | 2.84 | 2.89 |
| PRP | 0.12 | 0.12 | 0.12 | 0.13 | 0.13 | 0.14 | 0.16 | 0.16 | 0.17 | 0.17 | 0.14 | 0.03 | 0.15 | 0.16 | 0.17 | 0.16 | 0.15 | 0.17 | 0.18 | 0.15 | 0.14 |
| **Spain^b^** | **7.89** | **7.46** | **7.31** | **6.95** | **6.54** | **6.66** | **7.20** | **7.31** | **7.92** | **7.79** | **8.19** | **8.27** | **8.31** | **8.52** | **8.82** | **8.30** | **8.62** | **9.44** | **9.76** | **14.79** | **14.23** |
| NSP | 0.20 | 0.18 | 0.17 | 0.15 | 0.12 | 0.11 | 0.10 | 0.09 | 0.08 | 0.08 | 0.09 | 0.09 | 0.09 | 0.09 | 0.09 | 0.08 | 0.07 | 0.08 | 0.07 | 0.09 | 0.09 |
| ESP | 4.44 | 3.93 | 3.64 | 3.39 | 3.02 | 2.85 | 2.79 | 2.66 | 2.62 | 2.71 | 2.92 | 2.94 | 2.95 | 3.00 | 3.18 | 3.01 | 3.17 | 3.49 | 3.58 | 6.18 | 6.06 |
| COP | 2.97 | 3.08 | 3.24 | 3.16 | 3.14 | 3.46 | 4.07 | 4.35 | 5.02 | 4.80 | 4.99 | 5.06 | 5.08 | 5.24 | 5.34 | 5.02 | 5.19 | 5.66 | 5.92 | 8.30 | 7.85 |
| PRP | 0.28 | 0.28 | 0.26 | 0.26 | 0.26 | 0.25 | 0.24 | 0.21 | 0.20 | 0.20 | 0.19 | 0.19 | 0.19 | 0.20 | 0.20 | 0.19 | 0.19 | 0.21 | 0.19 | 0.23 | 0.23 |
| **Sweden** | **6.86** | **7.23** | **7.06** | **6.93** | **7.10** | **6.84** | **6.42** | **6.20** | **6.27** | **6.64** | **6.74** | **6.97** | **6.61** | **6.69** | **6.70** | **6.65** | **6.27** | **6.37** | **5.96** | **5.98** | **5.87** |
| NSP | 4.85 | 5.12 | 4.82 | 4.72 | 4.76 | 4.42 | 4.09 | 3.90 | 3.91 | 4.15 | 4.22 | 4.18 | 3.89 | 3.93 | 4.01 | 3.90 | 3.49 | 3.21 | 3.19 | 3.21 | 3.15 |
| ESP | 0.86 | 0.94 | 1.01 | 0.99 | 1.05 | 1.03 | 0.98 | 1.00 | 1.04 | 1.08 | 1.12 | 1.18 | 1.13 | 1.16 | 1.12 | 1.08 | 1.06 | 1.06 | 1.06 | 1.05 | 1.04 |
| COP | 0.16 | 0.17 | 0.16 | 0.14 | 0.15 | 0.15 | 0.13 | 0.13 | 0.15 | 0.16 | 0.17 | 0.17 | 0.16 | 0.16 | 0.16 | 0.16 | 0.16 | 0.54 | 0.17 | 0.18 | 0.20 |
| PRP | 0.99 | 1.00 | 1.08 | 1.08 | 1.15 | 1.23 | 1.21 | 1.18 | 1.17 | 1.25 | 1.24 | 1.45 | 1.43 | 1.43 | 1.42 | 1.51 | 1.56 | 1.56 | 1.54 | 1.53 | 1.48 |
| **United Kingdom** | **5.70** | **5.35** | **4.91** | **4.75** | **5.00** | **4.98** | **5.21** | **5.10** | **5.33** | **5.29** | **5.74** | **5.91** | **5.98** | **6.42** | **6.52** | **6.96** | **6.91** | **6.98** | **6.71** | **6.58** | **6.37** |
| NSP | 0.75 | 0.70 | 0.65 | 0.65 | 0.66 | 0.64 | 0.68 | 0.65 | 0.66 | 0.68 | 0.72 | 0.72 | 0.74 | 0.75 | 0.76 | 0.81 | 0.83 | 0.85 | 0.84 | 0.85 | 0.86 |
| ESP | 3.34 | 3.13 | 2.86 | 2.72 | 2.90 | 2.88 | 2.99 | 2.87 | 3.05 | 3.03 | 3.35 | 3.43 | 3.36 | 3.63 | 3.62 | 3.89 | 3.67 | 3.68 | 3.54 | 3.51 | 3.31 |
| COP | 1.03 | 0.91 | 0.76 | 0.68 | 0.67 | 0.66 | 0.68 | 0.68 | 0.70 | 0.61 | 0.66 | 0.70 | 0.74 | 0.80 | 0.83 | 0.89 | 0.88 | 0.88 | 0.81 | 0.71 | 0.70 |
| PRP | 0.58 | 0.61 | 0.65 | 0.70 | 0.77 | 0.81 | 0.86 | 0.90 | 0.91 | 0.98 | 1.02 | 1.07 | 1.14 | 1.23 | 1.30 | 1.37 | 1.52 | 1.57 | 1.51 | 1.51 | 1.51 |

Country, community consumption of penicillins (J01C); NSP, consumption of narrow-spectrum penicillins (J01CE); ESP, consumption of extended-spectrum penicillins (J01CA); COP, consumption of combination of penicillins, including β-lactamase inhibitors (J01CR); PRP, consumption of penicillinase-resistant penicillins (J01CF); **-**, no consumption reported; Numbers reported in *italic* are total care data, i.e. community and hospital sector combined; ^a^Data for Romania have a coverage in 2009 limited to 30-40%; ^b^Data for Spain include private prescriptions from 2016 onwards.

.

**Table S2. Consumption of penicillins (ATC J01C) in the community, expressed in packages per 1000 inhabitants per day, 23 EU/EEA countries, 2006-2017.**

| **Country** | **2006** | **2007** | **2008** | **2009** | **2010** | **2011** | **2012** | **2013** | **2014** | **2015** | **2016** | **2017** |  |
| --- | --- | --- | --- | --- | --- | --- | --- | --- | --- | --- | --- | --- | --- |
| **Austria** | - | **0.62** | **0.64** | **0.68** | **0.67** | **0.65** | **0.65** | **0.76** | **0.68** | **0.68** | **0.68** | **0.74** |  |
| NSP | - | 0.13 | 0.13 | 0.12 | 0.11 | 0.10 | 0.10 | 0.11 | 0.09 | 0.09 | 0.09 | 0.09 |  |
| ESP | - | 0.10 | 0.10 | 0.11 | 0.11 | 0.11 | 0.11 | 0.12 | 0.11 | 0.11 | 0.11 | 0.13 |  |
| COP | - | 0.39 | 0.41 | 0.45 | 0.45 | 0.44 | 0.45 | 0.53 | 0.47 | 0.48 | 0.47 | 0.52 |  |
| PRP | - | <0.01 | <0.01 | <0.01 | <0.01 | <0.01 | <0.01 | <0.01 | <0.01 | <0.01 | <0.01 | <0.01 |  |
| **Belgium^a^** | - | **1.17** | **1.28** | **1.25** | **1.29** | **1.27** | **1.27** | **1.17** | **1.13** | **1.08** | **1.39** | **1.27** |  |
| NSP | - | 0.02 | 0.02 | 0.01 | 0.01 | 0.01 | 0.01 | 0.01 | 0.01 | 0.01 | 0.01 | <0.01 |  |
| ESP | - | 0.56 | 0.62 | 0.57 | 0.61 | 0.60 | 0.60 | 0.60 | 0.55 | 0.56 | 0.63 | 0.58 |  |
| COP | - | 0.53 | 0.57 | 0.60 | 0.59 | 0.60 | 0.59 | 0.50 | 0.51 | 0.45 | 0.67 | 0.61 |  |
| PRP | - | 0.06 | 0.06 | 0.07 | 0.07 | 0.07 | 0.07 | 0.07 | 0.06 | 0.06 | 0.08 | 0.08 |  |
| **Bulgaria** | **1.18** | **1.20** | **1.15** | **0.98** | **0.92** | **0.87** | **0.81** | **0.87** | **0.78** | **0.75** | **0.69** | **0.72** |  |
| NSP | 0.26 | 0.23 | 0.18 | 0.14 | 0.12 | 0.04 | 0.03 | 0.03 | 0.02 | 0.02 | 0.02 | 0.02 |  |
| ESP | 0.67 | 0.69 | 0.67 | 0.53 | 0.51 | 0.52 | 0.48 | 0.50 | 0.44 | 0.41 | 0.38 | 0.38 |  |
| COP | 0.24 | 0.28 | 0.31 | 0.30 | 0.29 | 0.31 | 0.29 | 0.34 | 0.32 | 0.32 | 0.29 | 0.33 |  |
| PRP | - | - | - | - | - | - | - | - | - | - | - | - |  |
| **Croatia** | - | **1.33** | **1.30** | **1.11** | **1.00** | **0.99** | **1.11** | **1.13** | **1.15** | **1.15** | **1.11** | **1.09** |  |
| NSP | - | 0.15 | 0.14 | 0.12 | 0.10 | 0.09 | 0.08 | 0.08 | 0.07 | 0.05 | 0.06 | 0.06 |  |
| ESP | - | 0.62 | 0.56 | 0.47 | 0.37 | 0.33 | 0.32 | 0.34 | 0.34 | 0.34 | 0.31 | 0.28 |  |
| COP | - | 0.54 | 0.57 | 0.52 | 0.53 | 0.56 | 0.71 | 0.71 | 0.73 | 0.75 | 0.74 | 0.75 |  |
| PRP | - | 0.03 | 0.02 | - | - | - | <0.01 | <0.01 | <0.01 | <0.01 | <0.01 | <0.01 |  |
| **Czechia** | - | **0.69** | - | - | **0.98** | **1.02** | **0.80** | **0.91** | **0.96** | **0.94** | - | - |  |
| NSP | - | 0.26 | - | - | 0.25 | 0.26 | 0.18 | 0.23 | 0.23 | 0.22 | - | - |  |
| ESP | - | 0.16 | - | - | 0.14 | 0.15 | 0.14 | 0.15 | 0.14 | 0.13 | - | - |  |
| COP | - | 0.28 | - | - | 0.50 | 0.53 | 0.42 | 0.47 | 0.50 | 0.50 | - | - |  |
| PRP | - | \| - \| \| --- \| | - | - | 0.08 | 0.07 | 0.06 | 0.07 | 0.10 | 0.09 | - | - |  |
| **Denmark** | - | **1.18** | **1.15** | **1.13** | **1.19** | **1.20** | **1.11** | **1.12** | **1.10** | **1.08** | **1.06** | **1.03** |  |
| NSP | - | 0.68 | 0.65 | 0.61 | 0.63 | 0.63 | 0.56 | 0.55 | 0.52 | 0.49 | 0.47 | 0.45 |  |
| ESP | - | 0.36 | 0.36 | 0.36 | 0.38 | 0.38 | 0.35 | 0.35 | 0.35 | 0.35 | 0.35 | 0.35 |  |
| COP | - | 0.02 | 0.02 | 0.03 | 0.05 | 0.06 | 0.07 | 0.09 | 0.09 | 0.09 | 0.09 | 0.08 |  |
| PRP | - | 0.13 | 0.13 | 0.12 | 0.13 | 0.13 | 0.12 | 0.14 | 0.14 | 0.14 | 0.14 | 0.15 |  |
| Country, community consumption of penicillins (J01C); NSP, consumption of narrow-spectrum penicillins (J01CE); ESP, consumption of extended-spectrum penicillins (J01CA); COP, consumption of combination of penicillins, including β-lactamase inhibitors (J01CR); PRP, consumption of penicillinase-resistant penicillins (J01CF); **-**, no consumption reported; Numbers reported in *italic* are total care data, i.e. community and hospital sector combined; ^a^Data for Belgium are slightly overestimated from 2016 onwards (nursing homes counting units versus packages before 2016); ^b^Data for the Netherlands are based on average package size; ^c^Data for Spain include private prescriptions from 2016 onwards. | | | | | | | | | | | | | |
| **Estonia** | **0.69** | **0.74** | **0.71** | **0.65** | **0.62** | **0.66** | **0.63** | **0.61** | **0.59** | **0.59** | **0.57** | **0.57** |  |
| NSP | 0.08 | 0.09 | 0.09 | 0.07 | 0.07 | 0.07 | 0.07 | 0.06 | 0.06 | 0.05 | 0.06 | 0.06 |  |
| ESP | 0.49 | 0.50 | 0.46 | 0.43 | 0.39 | 0.40 | 0.38 | 0.35 | 0.32 | 0.30 | 0.27 | 0.25 |  |
| COP | 0.12 | 0.15 | 0.16 | 0.15 | 0.16 | 0.18 | 0.18 | 0.20 | 0.21 | 0.23 | 0.24 | 0.26 |  |
| PRP | <0.01 | <0.01 | <0.01 | <0.01 | <0.01 | <0.01 | \| - \| \| --- \| | <0.01 | <0.01 | <0.01 | \| - \| \| --- \| | \| - \| \| --- \| |  |
| **Finland** | - | - | **0.74** | **0.74** | **0.81** | **0.80** | **0.77** | **0.74** | **0.76** | **0.74** | **0.72** | **0.66** |  |
| NSP | - | - | 0.19 | 0.19 | 0.20 | 0.19 | 0.19 | 0.18 | 0.18 | 0.17 | 0.17 | 0.15 |  |
| ESP | - | - | 0.41 | 0.41 | 0.46 | 0.45 | 0.43 | 0.42 | 0.43 | 0.42 | 0.42 | 0.39 |  |
| COP | - | - | 0.13 | 0.13 | 0.15 | 0.15 | 0.15 | 0.14 | 0.15 | 0.14 | 0.13 | 0.11 |  |
| PRP | - | - | <0.01 | <0.01 | - | - | <0.01 | <0.01 | <0.01 | <0.01 | <0.01 | 0.01 |  |
| **France** | - | - | - | - | **2.29** | **2.38** | **2.47** | **2.59** | **2.52** | **2.61** | **2.73** | **2.66** |  |
| NSP | - | - | - | - | 0.04 | 0.05 | 0.04 | 0.05 | 0.05 | 0.04 | 0.05 | 0.05 |  |
| ESP | - | - | - | - | 1.36 | 1.46 | 1.53 | 1.66 | 1.62 | 1.73 | 1.85 | 1.86 |  |
| COP | - | - | - | - | 0.76 | 0.79 | 0.82 | 0.81 | 0.78 | 0.78 | 0.78 | 0.72 |  |
| PRP | - | - | - | - | 0.13 | 0.09 | 0.07 | 0.07 | 0.07 | 0.06 | 0.05 | 0.04 |  |
| **Greece** | ***2.53*** | ***2.59*** | ***2.59*** | **1.47** | ***2.18*** | **1.44** | **1.35** | **1.41** | **1.56** | **1.62** | **1.78** | **1.94** |  |
| NSP | *0.15* | *0.16* | *0.13* | 0.08 | *0.05* | 0.04 | 0.01 | <0.01 | 0.01 | 0.01 | 0.02 | 0.02 |  |
| ESP | *0.90* | *0.79* | *0.84* | 0.61 | *0.64* | 0.54 | 0.51 | 0.50 | 0.51 | 0.52 | 0.50 | 0.49 |  |
| COP | *1.46* | *1.62* | *1.60* | 0.78 | *1.47* | 0.85 | 0.83 | 0.91 | 1.04 | 1.09 | 1.25 | 1.43 |  |
| PRP | *0.02* | *0.02* | *0.02* | <0.01 | *0.03* | <0.01 | <0.01 | <0.01 | <0.01 | <0.01 | <0.01 | <0.01 |  |
| **Iceland** | - | - | - | - | ***1.45*** | ***1.45*** | ***1.43*** | ***1.39*** | **1.18** | **1.17** | **1.21** | **1.20** |  |
| NSP | - | - | - | - | *0.27* | *0.27* | *0.26* | *0.23* | 0.22 | 0.20 | 0.21 | 0.19 |  |
| ESP | - | - | - | - | *0.45* | *0.44* | *0.47* | *0.41* | 0.33 | 0.35 | 0.42 | 0.47 |  |
| COP | - | - | - | - | *0.51* | *0.51* | *0.49* | *0.55* | 0.48 | 0.49 | 0.44 | 0.41 |  |
| PRP | - | - | - | - | *0.23* | *0.23* | *0.21* | *0.20* | 0.15 | 0.14 | 0.14 | 0.13 |  |
| **Ireland** | - | **1.33** | - | - | **1.25** | **1.31** | **1.33** | **1.36** | **1.24** | **1.30** | **1.30** | **1.15** |  |
| NSP | - | 0.09 | - | - | 0.08 | 0.09 | 0.09 | 0.10 | 0.09 | 0.09 | 0.10 | 0.10 |  |
| ESP | - | 0.27 | - | - | 0.24 | 0.26 | 0.26 | 0.24 | 0.24 | 0.26 | 0.27 | 0.26 |  |
| COP | - | 0.90 | - | - | 0.86 | 0.90 | 0.91 | 0.93 | 0.81 | 0.86 | 0.85 | 0.70 |  |
| PRP | - | 0.06 | - | - | 0.07 | 0.06 | 0.07 | 0.09 | 0.10 | 0.10 | 0.08 | 0.09 |  |
| Country, community consumption of penicillins (J01C); NSP, consumption of narrow-spectrum penicillins (J01CE); ESP, consumption of extended-spectrum penicillins (J01CA); COP, consumption of combination of penicillins, including β-lactamase inhibitors (J01CR); PRP, consumption of penicillinase-resistant penicillins (J01CF); **-**, no consumption reported; Numbers reported in *italic* are total care data, i.e. community and hospital sector combined; ^a^Data for Belgium are slightly overestimated from 2016 onwards (nursing homes counting units versus packages before 2016); ^b^Data for the Netherlands are based on average package size; ^c^Data for Spain include private prescriptions from 2016 onwards. | | | | | | | | | | | | | |
| **Italy** | - | - | **1.70** | - | **1.63** | **1.62** | **1.59** | **1.65** | **1.58** | **1.56** | **1.53** | **1.37** |  |
| NSP | - | - | 0.02 | - | 0.02 | 0.01 | <0.01 | <0.01 | <0.01 | <0.01 | <0.01 | <0.01 |  |
| ESP | - | - | 0.63 | - | 0.52 | 0.48 | 0.46 | 0.44 | 0.40 | 0.38 | 0.37 | 0.31 |  |
| COP | - | - | 1.05 | - | 1.10 | 1.13 | 1.13 | 1.20 | 1.17 | 1.17 | 1.15 | 1.06 |  |
| PRP | - | - | <0.01 | - | <0.01 | <0.01 | <0.01 | <0.01 | <0.01 | <0.01 | <0.01 | <0.01 |  |
| **Latvia** | - | - | - | - | **0.66** | **0.73** | **0.73** | **0.76** | **0.71** | **0.75** | **0.75** | **0.81** |  |
| NSP | - | - | - | - | 0.01 | <0.01 | <0.01 | 0.02 | 0.03 | 0.03 | 0.03 | 0.04 |  |
| ESP | - | - | - | - | 0.46 | 0.53 | 0.51 | 0.52 | 0.47 | 0.49 | 0.48 | 0.51 |  |
| COP | - | - | - | - | 0.18 | 0.20 | 0.20 | 0.22 | 0.21 | 0.23 | 0.23 | 0.26 |  |
| PRP | - | - | - | - | <0.01 | \| - \| \| --- \| | 0.01 | <0.01 | <0.01 | <0.01 | <0.01 | <0.01 |  |
| **Lithuania** | - | ***1.41*** | ***1.38*** | ***1.11*** | ***1.03*** | ***1.10*** | **1.01** | **1.15** | **0.97** | **1.02** | **1.03** | **1.04** |  |
| NSP | - | *0.32* | *0.25* | *0.12* | *0.08* | *0.07* | 0.06 | 0.06 | 0.06 | 0.05 | 0.06 | 0.06 |  |
| ESP | - | *0.80* | *0.86* | *0.75* | *0.70* | *0.76* | 0.71 | 0.80 | 0.67 | 0.71 | 0.69 | 0.69 |  |
| COP | - | *0.29* | *0.26* | *0.24* | *0.25* | *0.26* | 0.25 | 0.29 | 0.24 | 0.26 | 0.29 | 0.29 |  |
| PRP | - | *<0.01* | *<0.01* | *<0.01* | *<0.01* | *-* | - | - | - | - | - | <0.01 |  |
| **Luxembourg** | **1.23** | **1.28** | **1.25** | **1.27** | **1.22** | **1.16** | **1.13** | **1.13** | **1.07** | **1.04** | **1.08** | - |  |
| NSP | 0.02 | 0.02 | 0.02 | 0.01 | 0.01 | 0.01 | <0.01 | <0.01 | <0.01 | <0.01 | <0.01 | - |  |
| ESP | 0.52 | 0.54 | 0.54 | 0.54 | 0.52 | 0.47 | 0.46 | 0.46 | 0.43 | 0.43 | 0.46 | - |  |
| COP | 0.64 | 0.67 | 0.64 | 0.67 | 0.65 | 0.64 | 0.63 | 0.63 | 0.60 | 0.56 | 0.59 | - |  |
| PRP | 0.05 | 0.05 | 0.05 | 0.05 | 0.05 | 0.04 | 0.04 | 0.04 | 0.04 | 0.04 | 0.04 | - |  |
| **Netherlands^b^** | - | - | **0.56** | **0.57** | - | - | - | - | - | - | - | - |  |
| NSP | - | - | 0.06 | 0.06 | - | - | - | - | - | - | - | - |  |
| ESP | - | - | 0.24 | 0.24 | - | - | - | - | - | - | - | - |  |
| COP | - | - | 0.19 | 0.20 | - | - | - | - | - | - | - | - |  |
| PRP | - | - | 0.08 | 0.08 | - | - | - | - | - | - | - | - |  |
| **Portugal** | - | - | **1.12** | **1.11** | **1.13** | **1.14** | **1.13** | **0.99** | **1.02** | **1.04** | **1.09** | **1.05** |  |
| NSP | - | - | 0.09 | 0.05 | 0.08 | 0.07 | 0.07 | 0.06 | 0.06 | 0.04 | 0.05 | 0.05 |  |
| ESP | - | - | 0.21 | 0.21 | 0.20 | 0.22 | 0.21 | 0.17 | 0.19 | 0.20 | 0.22 | 0.22 |  |
| COP | - | - | 0.73 | 0.75 | 0.75 | 0.77 | 0.76 | 0.68 | 0.70 | 0.73 | 0.74 | 0.71 |  |
| PRP | - | - | 0.10 | 0.10 | 0.10 | 0.08 | 0.09 | 0.08 | 0.08 | 0.08 | 0.08 | 0.07 |  |
| Country, community consumption of penicillins (J01C); NSP, consumption of narrow-spectrum penicillins (J01CE); ESP, consumption of extended-spectrum penicillins (J01CA); COP, consumption of combination of penicillins, including β-lactamase inhibitors (J01CR); PRP, consumption of penicillinase-resistant penicillins (J01CF); **-**, no consumption reported; Numbers reported in *italic* are total care data, i.e. community and hospital sector combined; ^a^Data for Belgium are slightly overestimated from 2016 onwards (nursing homes counting units versus packages before 2016); ^b^Data for the Netherlands are based on average package size; ^c^Data for Spain include private prescriptions from 2016 onwards. | | | | | | | | | | | | | |
| **Slovakia** | - | - | - | - | - | ***0.93*** | **0.76** | **0.85** | **0.77** | **0.83** | **0.78** | - |  |
| NSP | - | - | - | - | - | *0.18* | 0.16 | 0.17 | 0.15 | 0.15 | 0.14 | - |  |
| ESP | - | - | - | - | - | *0.16* | 0.14 | 0.16 | 0.12 | 0.12 | 0.11 | - |  |
| COP | - | - | - | - | - | *0.59* | 0.46 | 0.52 | 0.49 | 0.55 | 0.53 | - |  |
| PRP | - | - | - | - | - | *0.01* | - | - | - | - | - | - |  |
| **Slovenia** | - | **1.36** | **1.25** | **1.24** | **1.22** | **1.20** | **1.18** | **1.19** | **1.13** | **1.15** | **1.11** | **1.08** |  |
| NSP | - | 0.30 | 0.29 | 0.27 | 0.25 | 0.26 | 0.26 | 0.25 | 0.22 | 0.23 | 0.24 | 0.21 |  |
| ESP | - | 0.49 | 0.45 | 0.45 | 0.44 | 0.41 | 0.39 | 0.40 | 0.37 | 0.36 | 0.33 | 0.33 |  |
| COP | - | 0.51 | 0.50 | 0.49 | 0.50 | 0.51 | 0.51 | 0.52 | 0.53 | 0.54 | 0.51 | 0.52 |  |
| PRP | - | 0.07 | 0.01 | 0.02 | 0.02 | 0.02 | 0.02 | 0.02 | 0.02 | 0.02 | 0.02 | 0.02 |  |
| **Spain^c^** | - | - | - | - | **1.08** | **1.10** | **1.01** | **0.97** | **0.92** | **0.91** | **1.38** | **1.32** |  |
| NSP | - | - | - | - | 0.03 | 0.03 | 0.03 | 0.03 | 0.03 | 0.03 | 0.04 | 0.04 |  |
| ESP | - | - | - | - | 0.39 | 0.41 | 0.38 | 0.38 | 0.38 | 0.39 | 0.64 | 0.62 |  |
| COP | - | - | - | - | 0.62 | 0.61 | 0.55 | 0.53 | 0.48 | 0.46 | 0.67 | 0.63 |  |
| PRP | - | - | - | - | 0.05 | 0.04 | 0.04 | 0.04 | 0.03 | 0.03 | 0.04 | 0.03 |  |
| **Sweden** | - | - | - | **0.66** | **0.66** | **0.65** | **0.64** | **0.59** | **0.56** | **0.56** | **0.56** | **0.55** |  |
| NSP | - | - | - | 0.34 | 0.34 | 0.34 | 0.34 | 0.30 | 0.27 | 0.27 | 0.28 | 0.27 |  |
| ESP | - | - | - | 0.17 | 0.17 | 0.16 | 0.16 | 0.15 | 0.15 | 0.15 | 0.15 | 0.15 |  |
| COP | - | - | - | 0.02 | 0.02 | 0.02 | 0.02 | 0.02 | 0.02 | 0.02 | 0.02 | 0.02 |  |
| PRP | - | - | - | 0.14 | 0.13 | 0.13 | 0.13 | 0.12 | 0.12 | 0.12 | 0.12 | 0.11 |  |
| **United Kingdom** | - | - | - | - | - | - | - | - | - | **0.06** | - | - |  |
| NSP | - | - | - | - | - | - | - | - | - | 0.01 | - | - |  |
| ESP | - | - | - | - | - | - | - | - | - | 0.03 | - | - |  |
| COP | - | - | - | - | - | - | - | - | - | <0.01 | - | - |  |
| PRP | - | - | - | - | - | - | - | - | - | 0.01 | - | - |  |

Country, community consumption of penicillins (J01C); NSP, consumption of narrow-spectrum penicillins (J01CE); ESP, consumption of extended-spectrum penicillins (J01CA); COP, consumption of combination of penicillins, including β-lactamase inhibitors (J01CR); PRP, consumption of penicillinase-resistant penicillins (J01CF); **-**, no consumption reported; Numbers reported in *italic* are total care data, i.e. community and hospital sector combined; ^a^Data for Belgium are slightly overestimated from 2016 onwards (nursing homes counting units versus packages before 2016); ^b^Data for the Netherlands are based on average package size; ^c^Data for Spain include private prescriptions from 2016 onwards.

**
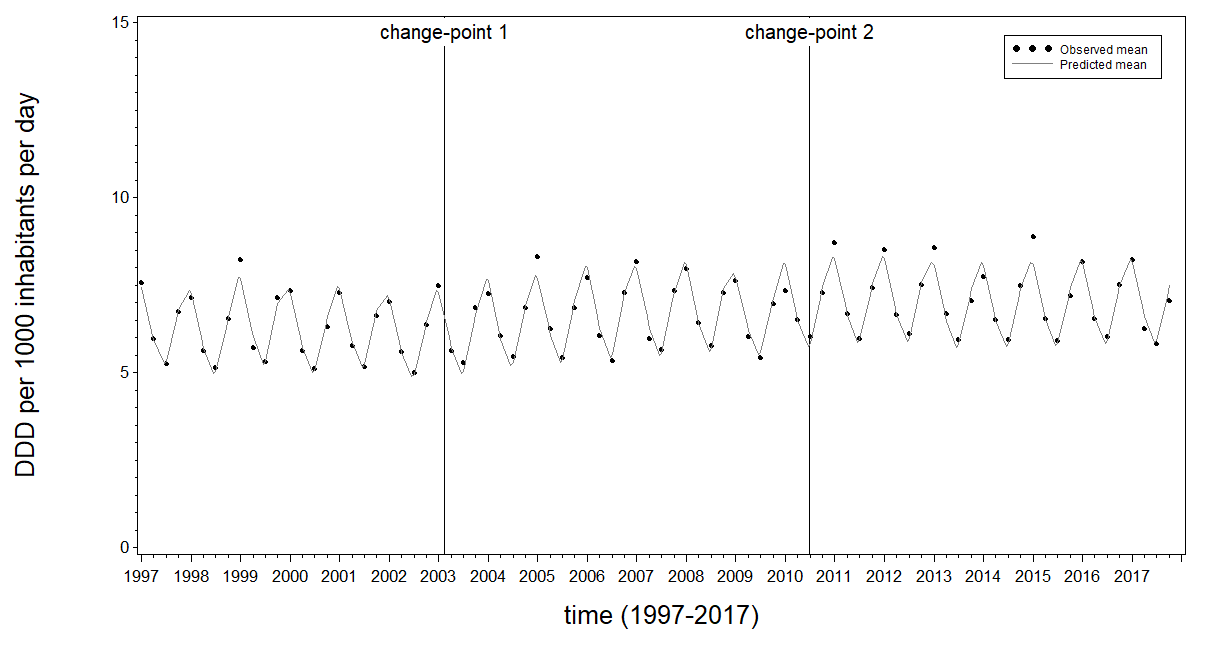
**

**Figure S1. Average of observed (dots) and predicted (solid line) consumption of penicillins (ATC J01C) in the community expressed in DDD (ATC/DDD index 2019) per 1000 inhabitants per day and based on quarterly data, 25 EU/EEA countries, 1997-2017.**

**
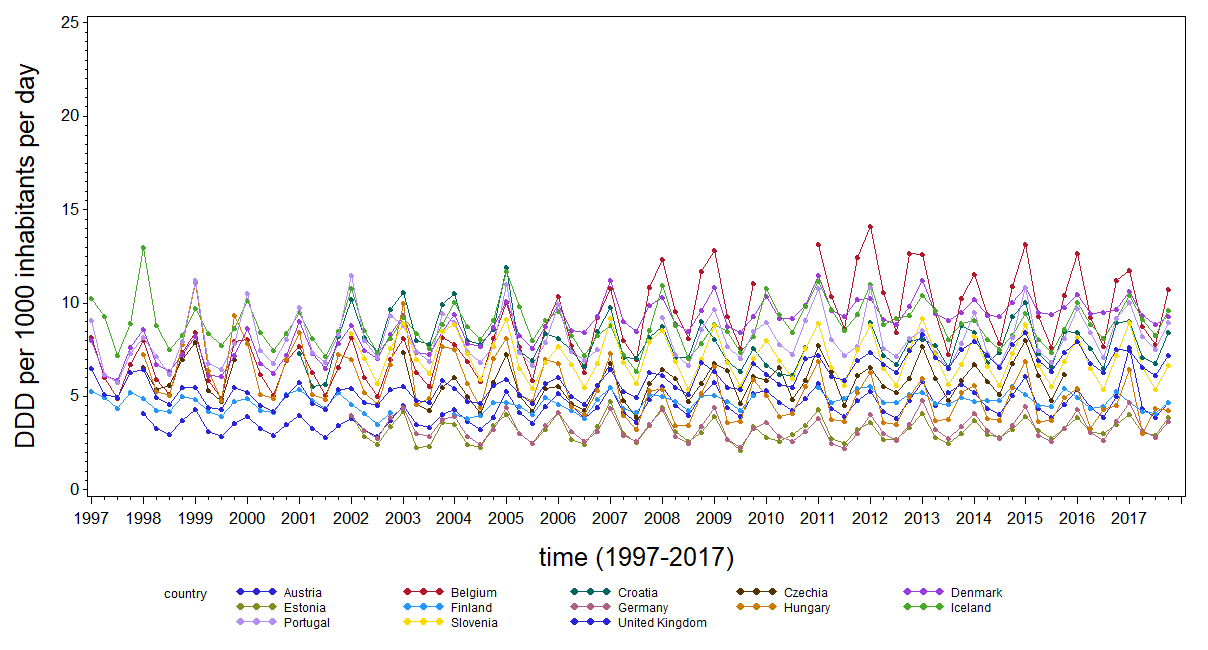
**

**Figure S2. Seasonal variation in consumption of penicillins (ATC J01C) in the community, expressed in DDD (ATC/DDD index 2019) per 1000 inhabitants per day, 13 EU/EEA countries reporting consumption per quarter for at least 15 years, 1997-2017.**


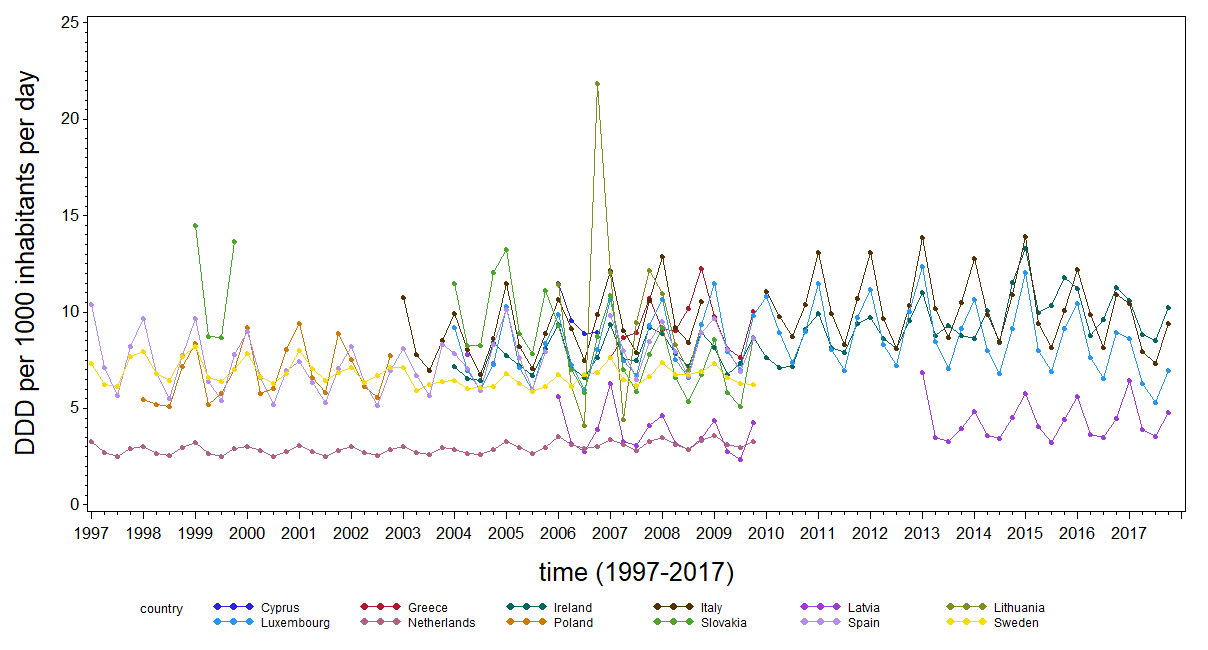


**Figure S3. Seasonal variation in consumption of penicillins (J01C) in the community, expressed in DDD (ATC/DDD index 2019) per 1000 inhabitants per day, 12 EU countries reporting consumption per quarter for less than 15 years, 1997-2017. For Cyprus, total care data, i.e. community and hospital sector combined, are used. For Spain, private prescriptions are included from 2016 onwards.**

^
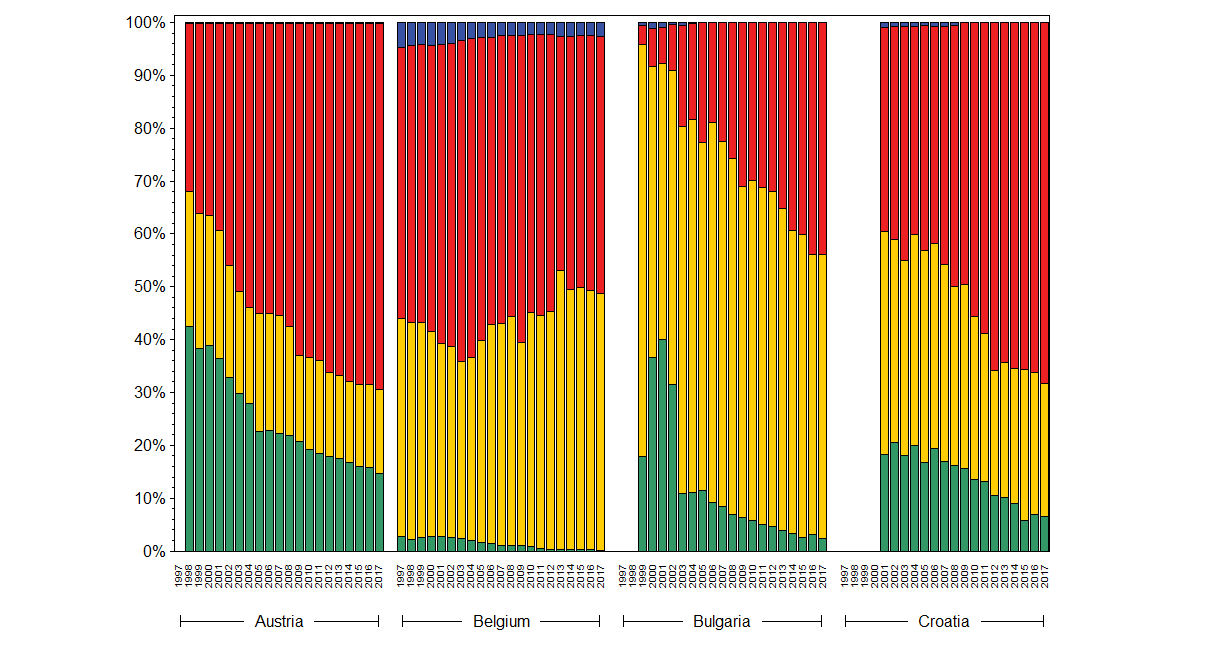
^

^
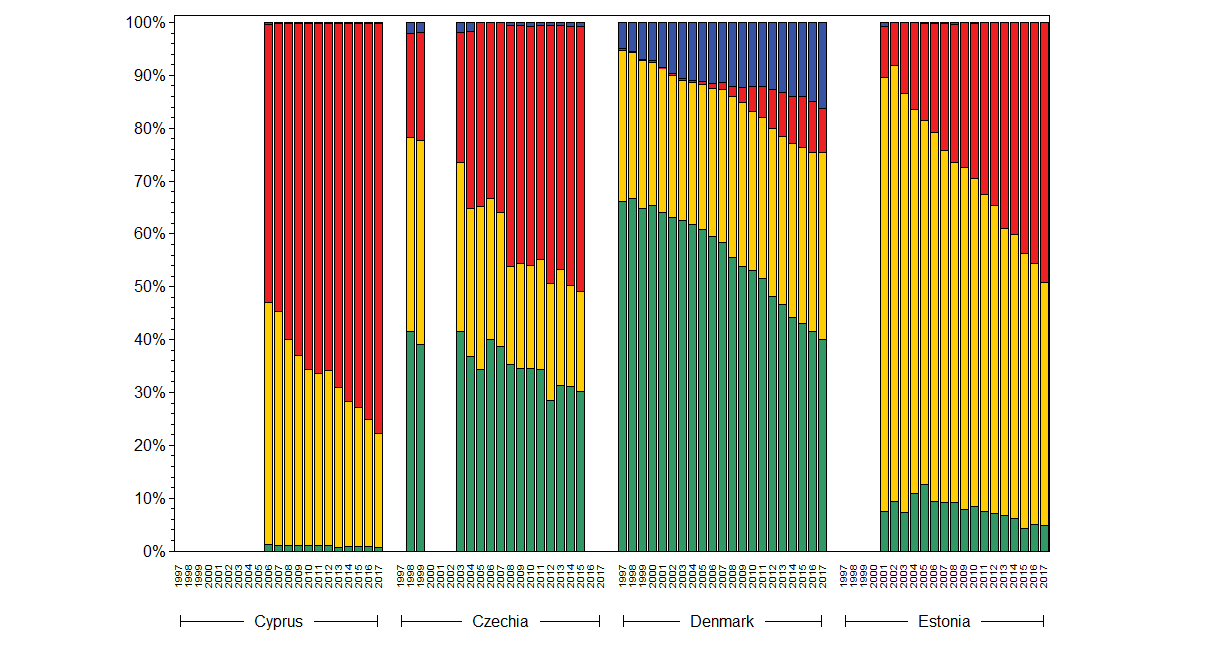
^

= narrow-spectrum penicillins (J01CE), = extended-spectrum penicillins (J01CA),
 = combinations of penicillins, incl. β-lactamase inhibitors (J01CR), = penicillinase-resistant penicillins (J01CF)

**Figure S4. Composition of penicillin (ATC J01C) consumption in the community, expressed in DDD (ATC/DDD index 2019) per 1000 inhabitants per day, 30 EU/EEA countries, 1997-2017. For Cyprus and Romania, total care data, i.e. community and hospital sector combined, are used. For Spain, private prescription are included from 2016 onwards. For Romania, data have a coverage in 2009 limited to 30-40%.**

^
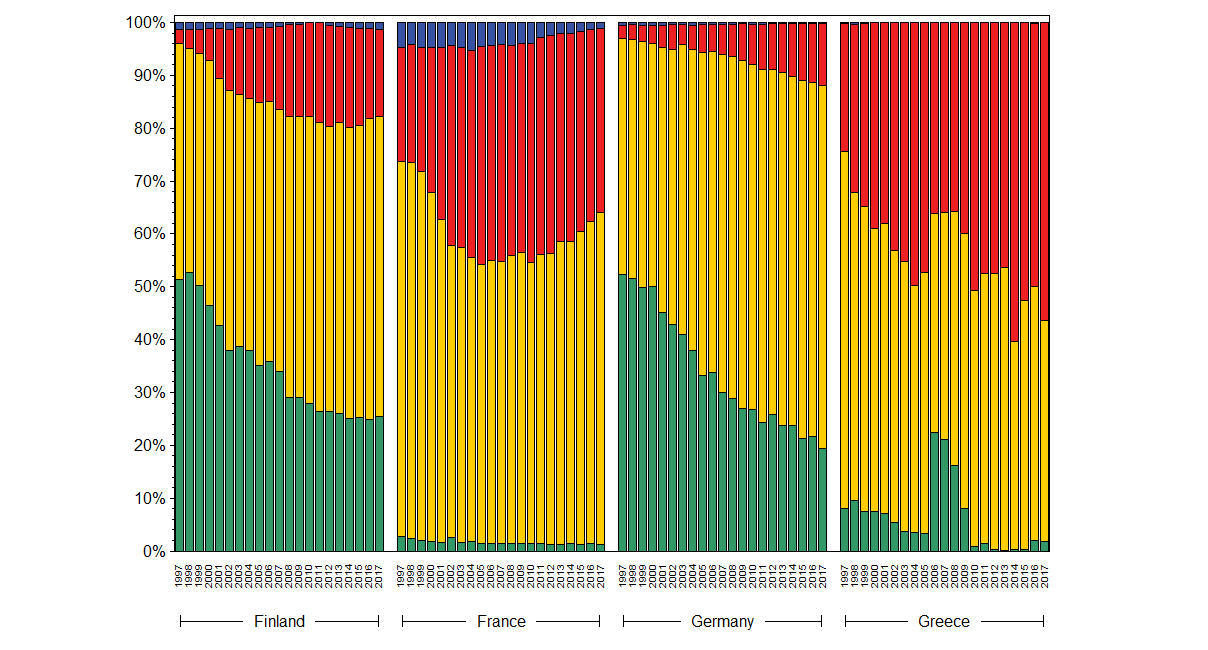
^

^
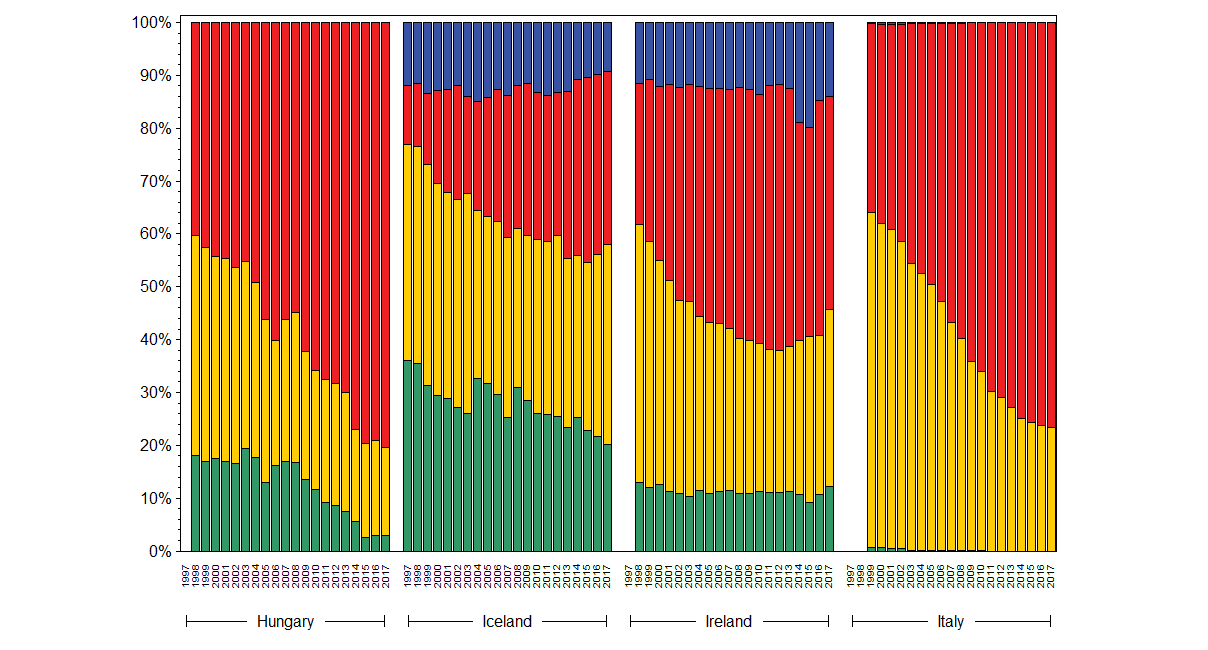
^

= narrow-spectrum penicillins (J01CE), = extended-spectrum penicillins (J01CA),
 = combinations of penicillins, incl. β-lactamase inhibitors (J01CR), = penicillinase-resistant penicillins (J01CF)

**Figure S4.** Continued

^
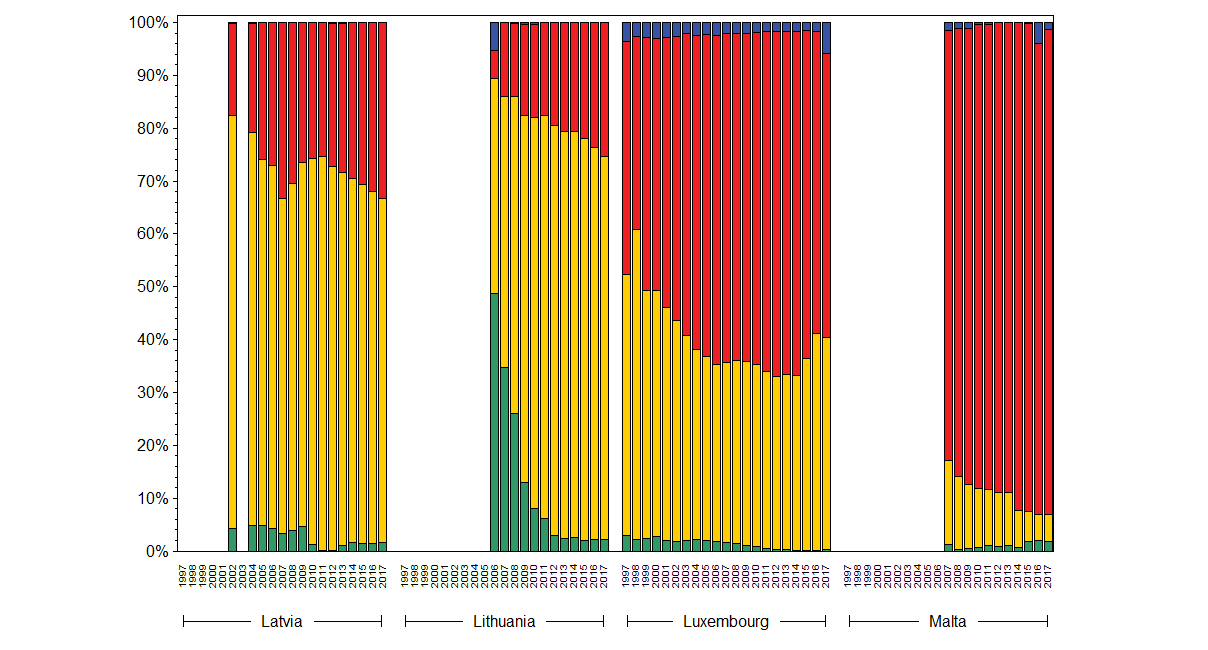
^

^
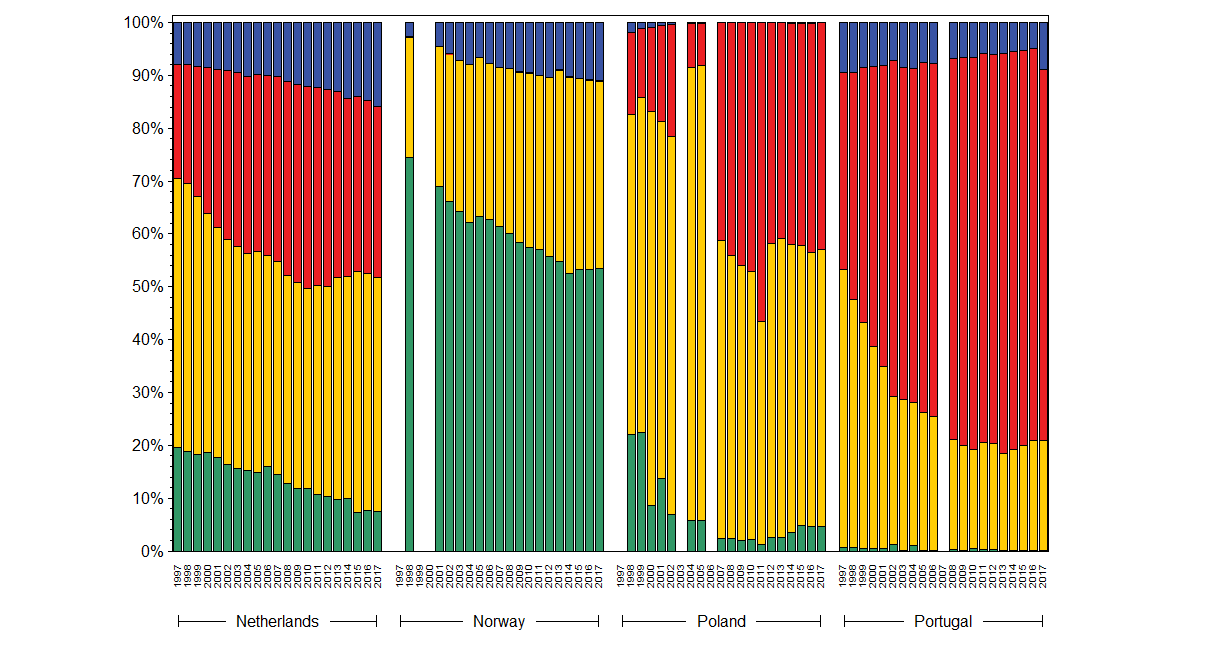
^

= narrow-spectrum penicillins (J01CE), = extended-spectrum penicillins (J01CA),
 = combinations of penicillins, incl. β-lactamase inhibitors (J01CR), = penicillinase-resistant penicillins (J01CF)

**Figure S4.** Continued

^
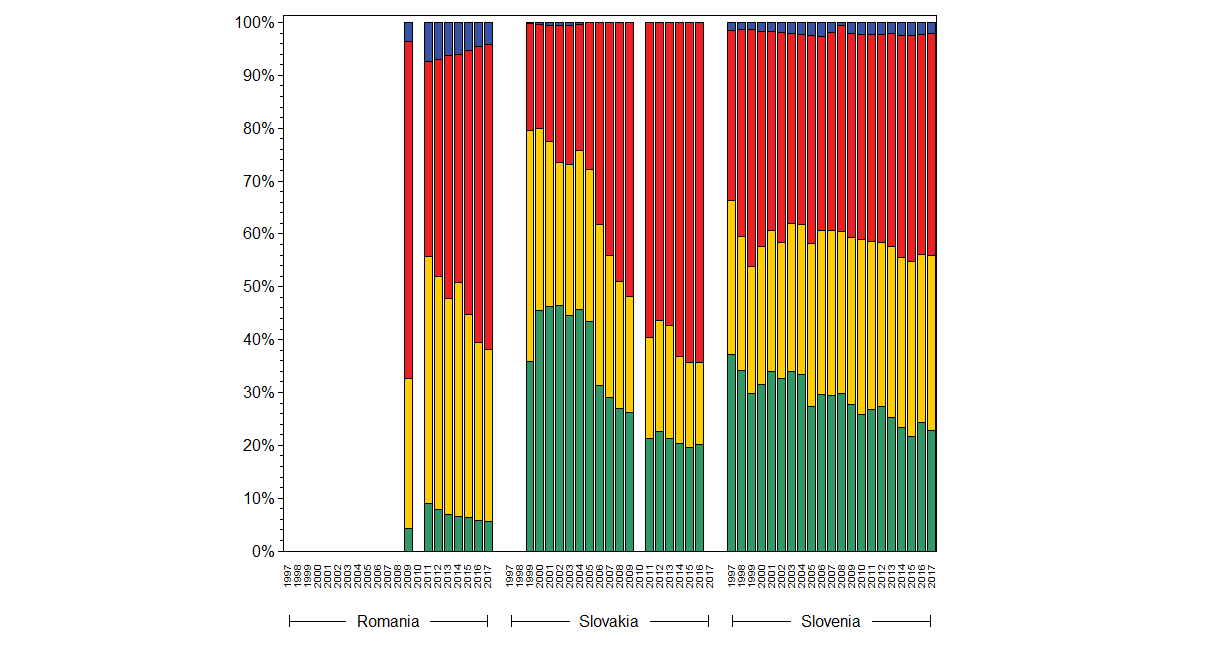
^


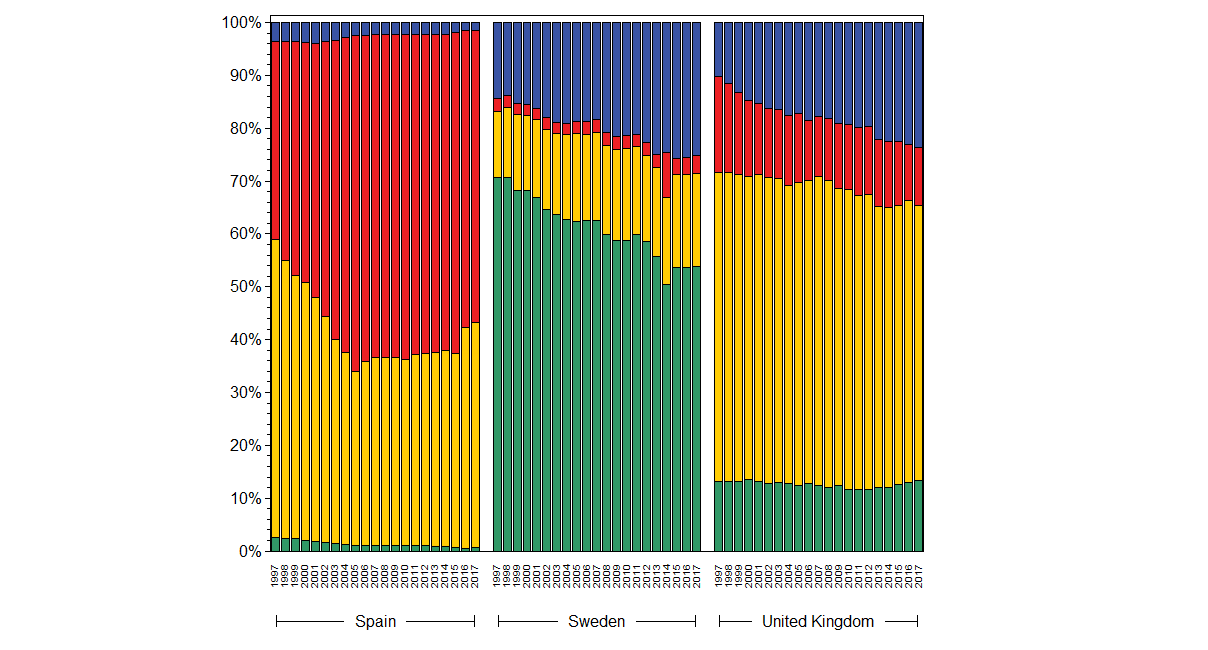


= narrow-spectrum penicillins (J01CE), = extended-spectrum penicillins (J01CA),
 = combinations of penicillins, incl. β-lactamase inhibitors (J01CR), = penicillinase-resistant penicillins (J01CF)

**Figure S4.** Continued
